# Safety and Immunogenicity of Pfs25-EPA/Alhydrogel^®^, a Transmission Blocking Vaccine against *Plasmodium falciparum*: An Open Label Study in Malaria Naïve Adults

**DOI:** 10.1371/journal.pone.0163144

**Published:** 2016-10-17

**Authors:** Kawsar R. Talaat, Ruth D. Ellis, Janet Hurd, Autumn Hentrich, Erin Gabriel, Noreen A. Hynes, Kelly M. Rausch, Daming Zhu, Olga Muratova, Raul Herrera, Charles Anderson, David Jones, Joan Aebig, Sarah Brockley, Nicholas J. MacDonald, Xiaowei Wang, Michael P. Fay, Sara A. Healy, Anna P. Durbin, David L. Narum, Yimin Wu, Patrick E. Duffy

**Affiliations:** 1 Center For Immunization Research, Johns Hopkins Bloomberg School of Public Health, Baltimore, Maryland, United States of America; 2 Laboratory of Malaria Immunology and Vaccinology, National Institute of Allergy and Infectious Diseases, National Institutes of Health, Rockville, Maryland, United States of America; 3 Biostatistical Research Branch, Division of Clinical Research, National Institute of Allergy and Infectious Diseases, National Institutes of Health, Rockville, Maryland, United States of America; 4 Division of Infectious Diseases, Department of Medicine, Johns Hopkins University School of Medicine, Baltimore, Maryland, United States of America; Public Health England, UNITED KINGDOM

## Abstract

Transmission-blocking vaccines (TBVs) that target sexual stage parasite development could be an integral part of measures for malaria elimination. Pfs25 is a leading TBV candidate, and previous studies conducted in animals demonstrated an improvement of its functional immunogenicity after conjugation to EPA, a recombinant, detoxified ExoProtein A from *Pseudomonas aeruginosa*. In this report, we describe results of an open-label, dose-escalating Phase 1 trial to assess the safety and immunogenicity of Pfs25-EPA conjugates formulated with Alhydrogel^®^. Thirty malaria-naïve healthy adults received up to four doses of the conjugate vaccine, with 8, 16, or 47 μg of conjugated Pfs25 mass, at 0, 2, 4, and 10 months. Vaccinations were generally well tolerated. The majority of solicited adverse events were mild in severity with pain at the injection site the most common complaint. Anemia was the most common laboratory abnormality, but was considered possibly related to the study in only a minority of cases. No vaccine-related serious adverse events occurred. The peak geometric mean anti-Pfs25 antibody level in the highest dose group was 88 (95% CI 53, 147) μg/mL two weeks after the 4^th^ vaccination, and declined to near baseline one year later. Antibody avidity increased over successive vaccinations. Transmission blocking activity demonstrated in a standard membrane feeding assay (SMFA) also increased from the second to the third dose, and correlated with antibody titer and, after the final dose, with antibody avidity. These results support the further evaluation of Pfs25-EPA/Alhydrogel^®^ in a malaria-endemic population.

## Introduction

Despite recent successes in reducing cases and deaths, malaria continues to be a significant cause of morbidity and mortality worldwide. In 2015, an estimated 214 million cases of malaria led to over 438,000 deaths [1]. Advances in diagnostics, vector control and antimalarial drugs have had huge impacts on disease burden, but an effective vaccine for malaria remains a major public health priority, especially with the emergence and spread of resistance to antimalarials and insecticides. An updated Malaria Vaccine Technology Roadmap outlined strategic goals to be achieved by 2030: 1) licensure of *Plasmodium falciparum* and *Plasmodium vivax* vaccines with protective efficacy of at least 75% against clinical malaria and 2) development of malaria vaccines that reduce transmission of the parasite [2]. The Roadmap retained its 2006 interim (landmark) goal of a first-generation vaccine against *P*. *falciparum* with greater than 50% efficacy against severe disease and death and duration of greater than one year [3]. Toward this landmark goal, one partially protective vaccine (RTS,S) has completed Phase 3 trials and received a positive scientific opinion from the European Medicines Agency in July 2015 for use outside of the European Union [4]. With efficacy in the range of 50% against clinical malaria, this vaccine is intended for use in combination with other interventions as it would not be sufficient to control malaria by itself [5, 6]. Longterm follow-up of vaccinated children shows a loss of protective efficacy and, with time, an increased risk of malaria [7]. The irradiated sporozoite vaccine has also been shown to be protective against malaria challenge in participants, though protection in the initial trials required 5 intravenous (IV) vaccinations [8], a route of administration that poses obstacles in light of current WHO requirements [9]. This vaccine is currently under trial in endemic populations, including in children [10].

Reducing malaria transmission can also be achieved by immunizing potential parasite carriers, and inducing antibodies that target the sexual stages of the parasite. When mosquitoes feed on vaccinated parasite carriers, the presence of antibodies in the blood meal will arrest sexual stage parasite development within the mosquitoes and prevent new human infections. Although a transmission blocking vaccine (TBV) does not immediately reduce the chance of malaria infection in the vaccinated individuals, the vaccinees may be protected through herd immunity [11]. Several potential TBV targets have been evaluated. Pfs25, a surface antigen of ookinetes in the mosquito stage of *P*. *falciparum*, has long been a lead candidate for a malaria transmission blocking vaccine [12]. However, the soluble recombinant form of Pfs25 is poorly immunogenic [13]. A previous trial of Pfs25 and the *P*. *vivax* orthologue, Pvs25, formulated with Montanide^®^ ISA 51 (a water-in-oil emulsion) was halted due to unexpected systemic adverse events including two cases of erythema nodosum in the Pvs25/ISA 51 arm [14]. The systemic adverse event was likely due to the combination of antigen and adjuvant, as it did not occur in the Pfs25/ISA 51 arm, and nor did it occur in a previous Phase 1 trial of Pvs25 formulated with Alhydrogel^®^. Participants who received 2 doses of Pfs25/ISA 51 had detectable antibody responses with varying levels of transmission reducing activity demonstrated in standard membrane feeding assays (SMFAs) [14].

In an effort to overcome the poor immunogenicity of Pfs25, we conjugated Pfs25 to a detoxified mutant recombinant *Pseudomonas aeruginosa* ExoProtein A (EPA)[15]. The conjugates induced significantly higher antibody responses than did un-conjugated Pfs25 in animal studies [13, 15, 16]. Recombinant EPA is not a component of any licensed vaccine, but has been extensively studied in a conjugated typhoid vaccine tested in children as young as 2 months old [17, 18], and a *Shigella* vaccine tested in children ages 1–7 years [19–21]. No safety issues have been identified to date in these trials [17–20]. A process was developed to manufacture the Pfs25-EPA conjugates in cGMP compliance and suitable for clinical trials [15].

In this paper we report a Phase 1 trial designed to assess the safety, immunogenicity, and transmission blocking activity of the malaria vaccine candidate Pfs25-EPA formulated with Alhydrogel^®^ in healthy malaria-naïve adults.

## Materials and Methods

The protocol for this trial and supporting CONSORT checklist are available as supporting information; see S1 and S2 Files, respectively.

### Study Design and Objectives

This open label Phase 1 trial was performed at the Center for Immunization Research of the Johns Hopkins Bloomberg School of Public Health in Baltimore, MD. The study was conducted under an investigational new drug application with the US Food and Drug Administration (BB-IND #14781). The protocol was approved by the Institutional Review Board (IRB) of the National Institute of Allergy and Infectious Diseases (NIAID) and the Western IRB, and trial identification number at ClinicalTrials.gov is NCT01434381. All participants gave written informed consent in order to participate in the study. The protocol was amended several times during the course of the study, most significantly to add additional doses of vaccine, as it was noted in a preliminary analysis that antibody responses progressively increased with successive vaccinations. The primary objective of this study was to assess the safety and immunogenicity of Pfs25-EPA/Alhydrogel^®^ in malaria naïve US adults. Secondary objectives were to determine functional antibody responses to the Pfs25 protein as measured by SMFAs.

### Participants

Healthy adults age 18–50 were recruited from the Baltimore, MD region and were screened for the absence of significant medical conditions. Exclusion criteria included prior history of malaria infection, recent use of antimalarial medication, or planned travel to a malaria-endemic area. Participants were required to be negative for human immunodeficiency virus, hepatitis B, and hepatitis C, as well as to have normal complete blood count and alanine aminotransferase (ALT).

### Interventions

Pfs25H is a *Pichia pastoris*-expressed hexa-His tagged recombinant Pfs25 with a molecular mass of 20,437 Daltons [22]. rEPA is an *E*. *coli*-expressed recombinant protein with molecular mass of 66,975 Daltons [13]. The Pfs25-EPA conjugate was produced by reaction between thiolated Pfs25H and maleimide-activated rEPA, followed by purification using size-exclusion chromatography [15]. Pfs25-EPA was subsequently formulated with Alhydrogel^®^, an aluminum hydroxide gel (Brenntag, Denmark), and filled so that each vial contained conjugates comprising of 78 μg/mL Pfs25H and 93 μg/mL rEPA, bound to 1600 μg/mL Alhydrogel^®^ in a volume of 0.8 mL. The Pfs25H, the rEPA, the Pfs25-EPA conjugates, and the final Pfs25-EPA/Alhydrogel^®^ vaccine were manufactured in cGMP compliance at the Walter Reed Army Institute of Research Bioproduction Facility. The biochemical and biophysical stabilities, including recognition by conformation-sensitive, transmission blocking monoclonal antibodies, of Pfs25H, rEPA, the Pfs25-EPA conjugates, and the final Pfs25-EPA/Alhydrogel^®^ vaccine were monitored annually during the trial until after the last vaccination. The potency of the final Pfs25-EPA/Alhydrogel^®^ vaccine was also monitored semiannually during the trial until after the last vaccination. All results indicated the conjugate and formulated vaccine were stable and were in compliance with the preset specifications.

Vaccines were given by intramuscular injection into the deltoid muscle in alternating arms. Shortly before vaccination, a study pharmacist withdrew the appropriate volume for the dose each participant was to receive. An injection volume of 0.1 mL (Group 1a, low dose) would deliver 8 μg Pfs25H (i.e., conjugates comprised of 8 μg Pfs25H, and 9 μg rEPA bound to 160 μg Alhydrogel^®^**)**; 0.2 mL (Group 1b, medium dose), 16 μg Pfs25H (conjugates comprised of 16 μg Pfs25H, 19 μg rEPA, bound to 320 μg Alhydrogel^®^**)**; and 0.6 mL (Group 2, high dose), 47 μg Pfs25H (conjugates comprised of 47 μg Pfs25H and 56 μg rEPA, bound to 960 μg Alhydrogel^®^**)**. The sample sizes for Groups 1a and 1b were for safety, prior to dose escalation to Group 2, which was sized for more intensive evaluation of immunogenicity and transmission reducing activity after successive vaccinations.

### Study Procedures

This study was designed as an open label, dose escalation study to examine the safety and immunogenicity of Pfs25-EPA/Alhydrogel^®^. Participants were sequentially enrolled into 3 groups: Group 1a received 2 injections of the low dose of vaccine (8 μg Pfs25H) and Group 1b received 2 injections of the medium dose of vaccine (16 μg Pfs25H), at 0 and 2 months; and Group 2 received 4 injections of the high dose of vaccine (47 μg Pfs25H) at 0, 2, 4, and 10 months. One of the high responders in Group 1a also received a 3rd injection of the low dose of vaccine (8 μg Pfs25H) at 10 months.

On study day 0, each participant received their first dose of vaccine after eligibility was verified, and was observed for 30 minutes afterwards to ensure no immediate adverse events took place. Participants were then followed 3, 7, 14 and 28 days after each vaccination. Clinical evaluations were performed at each visit, and safety labs (CBC, ALT, creatinine) were conducted prior to and at days 3, 7, and 14 after each vaccination. Urine dipsticks for blood and protein were conducted prior to and 7, 14, and 28 days after each vaccination. For female participants, urine pregnancy tests were done at screening, prior to and 14 days after each vaccination. Participants were asked to keep a memory card documenting any adverse symptoms for 1 week after each vaccination. The original study design called for participants in Groups 1a and 1b to receive two doses of vaccine two months apart, and for participants in Group 2 to receive three doses of vaccine at months 0, 2, and 4. After initial results were reviewed, the protocol was amended and participants in Group 2 and one participant in Group 1a with high antibody responses were given an additional booster dose at approximately study day 300. Participants were followed 12 months after the last vaccination. Participants with higher antibody titers were invited to sign a separate consent for a large volume blood draw for serum if they otherwise met criteria.

Pre-scheduled safety reviews by an independent Safety Monitoring Committee (SMC) oversaw adverse event data as the study progressed.

### Antibody Titers

Antibody responses to vaccine were measured on plasma or sera obtained from the participants by a standard enzyme-linked immunosorbent assay (ELISA) as described previously [23]. Briefly, ELISA plates were coated with recombinant Pfs25H or rEPA. Plasma or sera collected from volunteers were tested against a set of serially diluted reference standard serum. The reference standard serum was generated by pooling sera from four volunteers after the booster vaccination. These reference standards were assigned ELISA unit (EU) values equal to the reciprocal of the dilution giving an optical density at 405 nm (OD_450_) of 1. Absorbance of the set of serially diluted reference standards was fitted to a 4-parameter hyperbolic function to generate a standard curve. Using these standard curves, the absorbance in anti-Pfs25 ELISA and anti-EPA ELISA of an individual test serum were converted to antibody unit values. The ELISA results were converted to μg/mL by multiplying a conversion factor (1 EU = 0.104 μg anti-Pfs25-specific IgG) obtained using the same methods described in Cheru et al [24].

### Immunofluorescence Assays

For surface labeling of live parasites, a previously reported procedure [25, 26] was followed with a few modifications. In brief, gametocyte-enriched NF54 parasites were allowed to exflagellate and fertilize by incubating in RPMI 1640 (KD Medical, Columbia, MD) containing 10% human serum for 90 min at room temperature. At the end of this incubation, parasites were brought to a final 1:500 dilution of sera collected on days 0, 314, and 356 from study volunteers and allowed to incubate for 30 minutes at room temperature. After washing thrice, the parasite suspension was spotted on glass slides, air dried and stored at -80°C. Following thawing and methanol-fixation, the slides were incubated with a mixture of mouse anti-Pfs25 mAbs 1G2 and 4B7 (diluted in PBS, 10% fetal bovine serum) at a final concentration of 20 μg/ml each for 30 min. The slides were then washed extensively with PBS. Finally, the slides were incubated with a mix of Alexa Fluor 488-conjugated goat anti-human IgG and Alexa Fluor 594-conjugated goat anti-mouse IgG (Invitrogen, Eugene, OR) at 20 μg/ml each for 30 min. After washing, the slides were mounted with coverslips using Vectashield mounting medium containing DAPI (4',6-diamidino-2-phenylindole; Vector Laboratories, Burlingame, CA). Images were acquired using an Olympus BX51 fluorescence microscope equipped with an Olympus DP72 microscope digital camera (Olympus, Melville, NY).

For recognition of Pfs25 on ookinetes by immune sera, *A*. *stephensi* mosquitoes (Nijmegen strain) were membrane-fed with an NF54 culture containing mature gametocytes. Mosquitoes were dissected 20 hours post feed and a thin blood smear was prepared from each mosquito midgut. The slides with smears were air dried and placed into sealed plastic bags with desiccant at 4°C for up to 2 weeks. On the day of the assay, slides were fixed with 90% acetone/10% methanol solution at -20°C for 10 min, followed by blocking with 5% milk/PBS. The slides were first incubated with Day 0, Day 314, or Day 356 sera from selected volunteers, at 1:500 and 1:1,000 dilutions for one hour, then with mAb 4B7 at a final concentration of 0.5 μg/mL for one hour, and finally with a mixture of Fluorescein conjugated goat IgG fraction to human IgG (MP Biomedicals, Santa Ann, CA) and Alexa Fluor 594 goat anti-mouse IgG (Invitrogen, Eugene, OR) at a final concentration of 10 μg/mL each for one hour. All antibody dilutions were made with 1%BSA in PBS, and all antibody incubations were followed by extensive washing with PBS. The slides were mounted with coverslips using Vectashield mounting medium containing DAPI. Images were acquired described above.

### Transmission Blocking and Reducing Activity

Transmission blocking activity (TBA, reduction in infection prevalence) and transmission reducing activity (TRA, reduction in infection intensity) of the sera were tested by an *ex vivo* standard membrane feeding assay (SMFA) as described previously [14]. Briefly, an in vitro 14–16 day-old gametocyte culture of *P*. *falciparum* (NF54 line) was evaluated for Stage V gametocytes (>0.5%) and the presence of exflagellation centers observed at 400X magnification (>1 per field). The culture was diluted with washed O+ RBCs from malaria naïve volunteers (Interstate Blood Bank, Memphis, Tennessee) to achieve 0.12% ± 0.05% concentration of Stage V gametocytes. For each sample, one hundred microliters (100 μL) of the pelleted diluted culture (100% haematocrit) was mixed with 160 μL of test serum and immediately fed to pre-starved (24–30 hours) 3–8 days old *Anopheles stephensi* (Nijmegen strain) mosquitoes through a Parafilm^®^ membrane stretched across a glass mosquito feeder (Chemglass CG-1835-70), kept warm by a jacket with 40°C circulating water. Test sera were not heat-inactivated. After the feed mosquitoes were kept for 8 days at 27°C and 80% humidity conditions to allow parasites to develop. Infectivity was measured by dissecting at least 20 mosquitoes per sample, staining the midguts with 0.05% mercurochrome solution in water for at least 20 min and counting the number of oocysts in each midgut. The feeding experiment was not analyzed unless the average oocyst count in the (at least) 20 dissected mosquitoes fed with naïve heat inactivated human serum pool (assay control) was more than four. The TBA and TRA are calculated by the following formulas:

$$TRA=100*\left(\frac{Mean\;Oocyst\;Number_{neg\;ctrl}-Mean\;Oocyst\;Number_{test}}{Mean\;Oocyst\;Number_{neg\;ctrl}}\right)$$

and

$$TBA=100*\left(\frac{Mean\;No.\;Inf.Mosquito_{neg\;ctrl}-Mean\;No.Inf.Mosquito_{test}}{Mean\;No.Inf.Mosquito_{neg\;ctrl}}\right)$$

where the negative control (neg ctrl) feed used pre-vaccination sera from the same volunteer. Each sample was tested in two independent feeding experiments, and these two TRA values were averaged to obtain a single subject level TRA for a given time point.

### Antibody Avidity

Antibody avidity was evaluated by a urea-displacement ELISA. Briefly, the test sera were first empirically adjusted to an appropriate concentration to provide a reference point, approximately 1.5–2 EU, to ensure the sample concentration after the urea wash remained within the linear range of the standard curve. The adjusted test sera were tested by ELISA following a wash with ascending concentrations of urea at 0, 2, 4, 6, 8, and 9 M. Antibody retention after the urea wash was calculated by dividing measured OD_450_ for each serum at each ascending urea concentration with measured OD_450_ at the reference point, i.e. 0 M of urea of the same serum. Avidity Index was expressed as slope of change of antibody retention for each serum at the ascending concentration of urea wash, integrating all data points for each test serum.

### Statistical Analyses

The safety analysis was descriptive and run in R [27]. All participants enrolled in the study were included in the safety analysis. Adverse events were either solicited (local and systemic events that were queried), laboratory, or unsolicited. They were assessed by their nature and timing to be related or unrelated to vaccine. Adverse events were reported by dosage grouping and by vaccination.

ELISA levels before and after given vaccinations were compared using Wilcoxon signed rank or rank sum tests, depending on whether the results were paired or not, respectively. Anti-EPA and Anti-Pfs25 ELISA titer decline from peak time point post vaccination were compared by Wilcoxon signed rank test on the slopes of log ELISA levels. Reported correlations between ELISA levels are Spearman rank correlations, which reduce the influence of outliers.

For SMFA, mean values of TRA from two replica assays on individual samples were presented. Standard deviation (SD) for an individual sample was calculated on all replicate tests available for a given time point. For estimation of EC_50_, i.e. the serum anti-Pfs25 level conferring 50% TRA, we fit a linear generalized estimating equation (GEE) model assuming Gaussian errors with the response being the log of the average of the oocyst count ratios (test/control) from the SMFAs. The expected responses are modeled with a simple 2 parameter linear model on the square root of the concurrent ELISA values, where each subject has 3 responses and 3 corresponding ELISA values measured on Days 70, 134, and 314 sera, representing the peak time points post vaccinations. We then back solved for the value of EC_50_. We calculated a 95% CI for this value using both the delta method and using Bootstrap; as both gave very similar results, the bootstrap method CI is reported.

Avidity indices are the estimated slopes of log OD_450_, representing retained anti-Pfs25 antibodies in individual sera after washes with ascending concentrations of urea. A Wilcoxon signed rank test was used to compare the avidity indices after successive vaccinations. Linear regression was used to investigate associations between TRA and avidity index, adjusted for ELISA level, for each peak post vaccination sera: D70, D134, D314.

## Results

### Study participants

**Fig 1:**
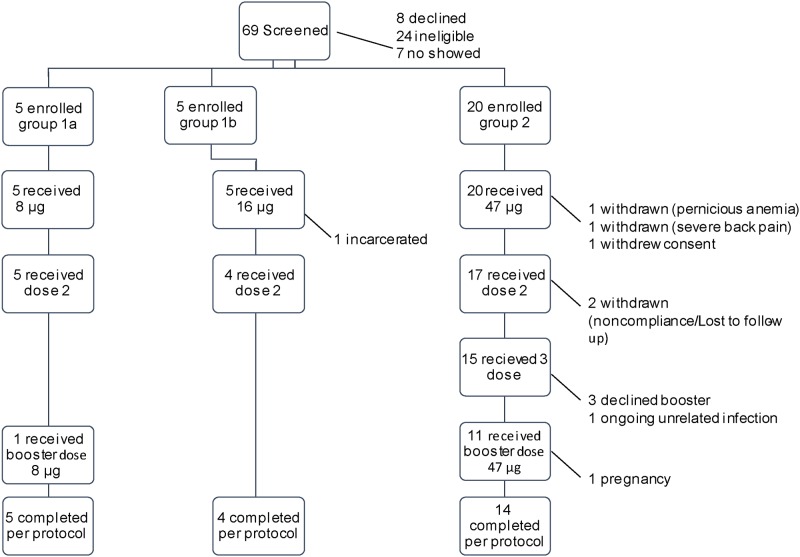
Study flow chart. Thirty (30) participants were enrolled, 23 completed the study per protocol.

Sixty-nine participants were screened for the study, of whom 30 were found to be eligible and enrolled in the study. The study flow chart is shown in Fig 1. Thirty participants (14 female) received at least 1 dose of the vaccine: 5 in the low dose group, 5 in the medium dose group, and 20 in the high dose group. Reflecting the population of East Baltimore, the participants were predominantly African American (80%), with 14% self-identifying as white, 3% as black/Hispanic and 3% as multiracial. The average age at first vaccination was 34 (range 23–50). Participants were enrolled in a sequential fashion to ensure that the lower dose of vaccine was tolerated before a higher dose was administered. The first doses were administered October 2011, and the final doses August 2012.

As shown in Fig 1, 5 participants in the low dose (8 μg of Pfs25) group received 2 doses of the vaccine; one of these participants had high titer antibody responses to the vaccine, and received a third, booster dose 8 months after her second dose. Five participants received 1 vaccination with the middle dose (16 μg of Pfs25), 4 of who received a second vaccination. The fifth withdrew from the study due to incarceration. Twenty participants received the first vaccination of the conjugate vaccine containing 47 μg of Pfs25H (high dose). One participant withdrew consent due to scheduling conflicts, and 2 were ineligible to continue (one because of unrelated ongoing severe lumbosacral back pain and a second for preexisting (undisclosed) pernicious anemia), consequently 17 participants received the second vaccination in this group. Two participants were subsequently withdrawn due to lack of adherence to the protocol requirements, resulting in 15 participants received the third vaccination at month 4. Of these 15 participants, 11 were interested and available to receive the booster 6 months later. One final participant was withdrawn from the study 6 months after the booster vaccination because of pregnancy. This participant was followed for safety. In all, 23/30 participants completed the study according to protocol.

### Safety

**Fig 2:**
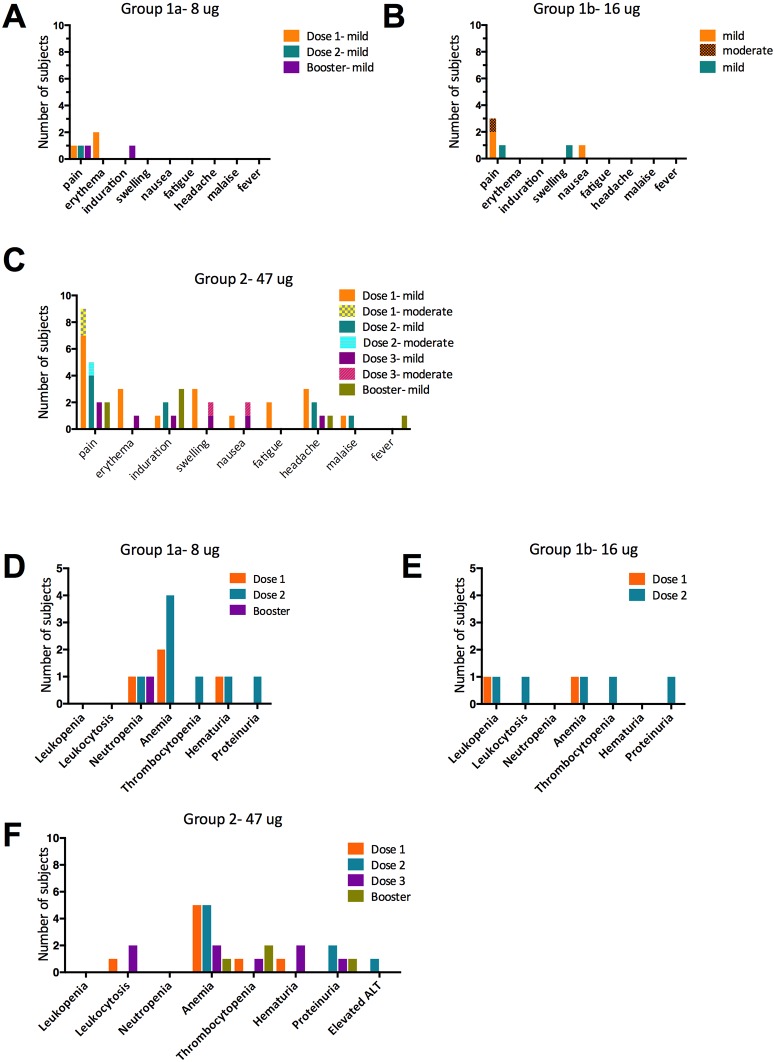
Summary of adverse events after each vaccination. Clinical solicited (A-C) and Laboratory (D-F) adverse events after each vaccination with each dose group are reported in individual graphs: Group 1a (A and D), 1b (B and E) and Group 2 (C and F). All adverse events were mild except when indicated as moderate.

The vaccine was well tolerated (Fig 2). Only a minority of participants in any dose group reported local or systemic symptoms, and the majority of adverse events were mild in nature (Fig 2A–2C). The most commonly reported symptom was pain at the injection site, reported in a total of 13 participants (43%) after the first vaccination, 10 (77% of 13) who had mild symptoms and 3 (23% of 13) who complained of moderate symptoms. Seven participants (27%) had pain after the second vaccination, 1 of which was moderate. Two (13%) participants had pain after the third dose and 3 (25%) after the booster dose. Headache was the most commonly reported systemic adverse event, occurring in 3 (10%) subjects after the first dose, 2 (8%) subjects after the second dose, and 1 subject after either the 3^rd^ or booster dose (Fig 2). Two serious adverse events (SAEs) occurred in 1 participant who was hospitalized twice for intractable lumbosacral back pain and radiculopathy (an exacerbation of a chronic preexisting condition). Both were deemed unrelated to the vaccination; the participant was withdrawn from the active portion of the study, and followed for safety.

Several laboratory abnormalities occurred during this study (Fig 2D–2F). The majority were mild and considered unrelated to vaccination, occurring in participants with histories of anemia, thrombocytopenia, and low baseline neutrophil counts. Anemia was the most common laboratory abnormality, but was considered possibly related to the study in only a minority of cases. There was no correlation between the vaccine dose and the rate or severity of anemia. Hematuria was seen in several women, and was considered unrelated to vaccination. No potentially immune-mediated or clinically significant dermatologic adverse events occurred.

### Immunogenicity

**Fig 3:**
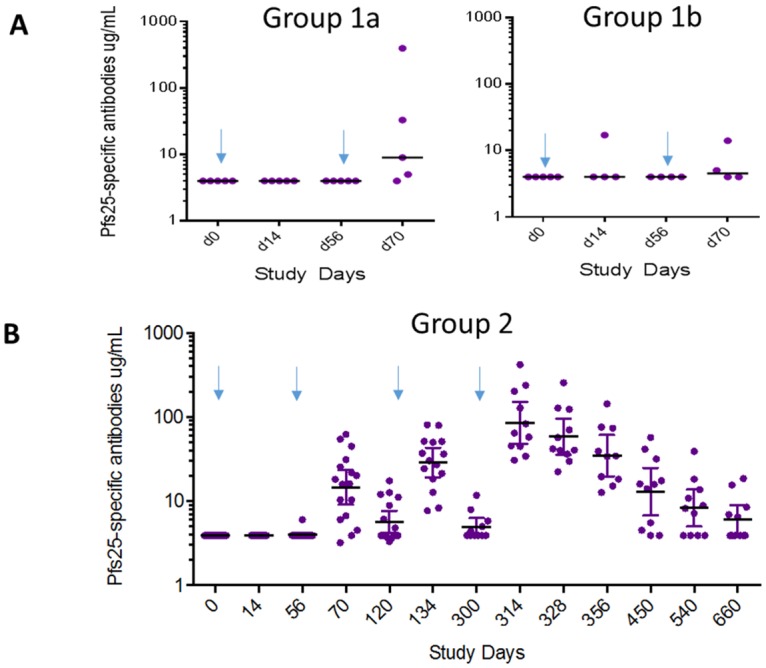
Anti-Pfs25 responses. A, Groups 1a and 1b participants, receiving conjugated proteins comprising of 8 μg and 16 μg Pfs25H, respectively. B, Group 2 participants, receiving 47 μg Pfs25H. Arrows indicate the day of vaccination. Closed circles represent antibody level and individual participants, and black bars indicate geometric mean of antibody levels and t-distribution 95% confidence interval (CI).

As shown in Fig 3A, only 1 participant in Groups 1a and1b, designated to receive 8 μg or 16 μg of Pfs25H respectively, developed detectable anti-Pfs25 antibody responses after first vaccination. The sero-conversion rate increased after the second vaccination on Day 56, but only one participant in Group 1a developed high antibody responses, with serum anti-Pfs25 concentration of 397 μg/mL, on Day 70, two weeks post the vaccination. The antibody level of this participant waned over time to near baseline on Day 300 when a booster dose of 8 μg of Pfs25H as part of the conjugated protein was given.

**Fig 4:**
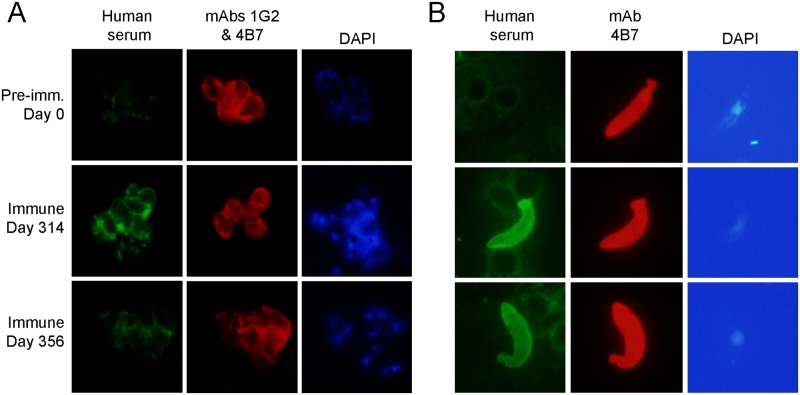
Immunofluorescence assays with immune sera. A. Surface labeling of zygotes with sera from one volunteer (#20) collected on days 0, 314 and 356, with Pfs25 specific mouse mAbs 1G2 and 4B7. B. Recognition of parasite protein in fixed ookinetes with sera from one volunteer (#20) collected on days 0, 314 and 356, with Pfs25 specific mouse mAb 4B7. Magnification 1000X.

The antibody responses after each successive vaccination in Group 2 participants are shown in Fig 3B. No antibody responses were detected two weeks after the first vaccination, though the proportion of antibody levels in responders increased after the second and third vaccinations on Days 56 and 120. The antibody levels increased further two weeks after the final booster dose on Day 300, with a peak geometric mean for anti-Pfs25 IgG level of 88 (95% CI 50–158) μg/mL on Day 314, then declined rapidly to 36 (95% CI 20–64) μg/mL on Day 356. The immune sera from these participants recognized zygotes and ookinetes (Fig 4) in same patterns as did anti-Pfs25 monoclonal antibodies; though as expected, the IFA titers of Day 356 sera seemed to be lower than the Day 314 sera (S1 Table). By Day 660, one year after the last vaccination and the end of the scheduled follow-up, the antibody titers were near the baseline level, as there was no significant difference in anti-Pfs25 IgG levels between Day 0 and Day 660 (*P-value* = 0.06, paired Wilcoxon test).

We also analyzed antibody responses induced by the conjugated rEPA, the carrier in the vaccine. There were no detectable anti-EPA antibodies in the participants prior to the vaccination. In contrast to anti-Pfs25 levels, however, over 40% of the participants developed anti-EPA antibodies after the first dose, and anti-EPA titer increases further with succession of vaccination. There was a moderate positive correlation between the levels of anti-Pfs25 and anti-EPA in individual subjects at peak post-vaccination times on days 70, 134 and 314 (Spearman rank correlation R = 0.42 bootstrap 95% CI (0.08, 0.66, 1-sided bootstrap P-value = 0.01). The rate of decline in anti-EPA levels from peak, however, was significantly slower than that of anti-Pfs25, as indicated in Wilcoxon signed rank test comparing subject specific slopes from day 314 to day 660 for anti-EPA to anti-Pfs25 (P-value = 0.01).

### Transmission reducing activity (TRA) of the immune sera

**Fig 5:**
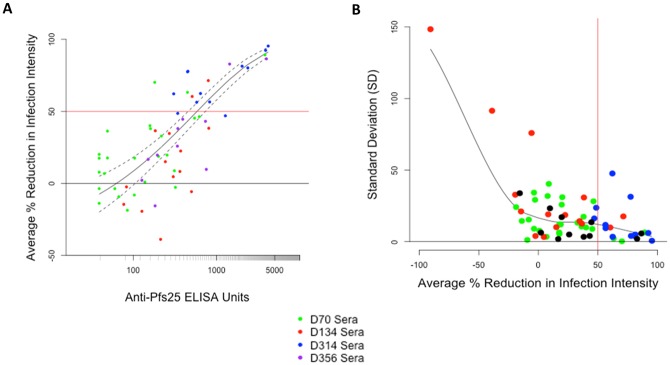
Transmisison reducing activity of immune sera. A. Transmission reducing activity of sera collected 2 weeks after each initial vaccination and 8 weeks after the booster vaccination. Each data point represents the average of two results in the SMFA, and lines indicate estimate EC50 and point-wise confidence interval by GEE model. B. Standard deviation (SD) of individual TRA from replica assays. Thick blue line indicates the threshold representing significant biological activity (50% TRA), the black line is a standard loess smooth curve. The green, red, blue, and black dots correspond to the data points on D70, D134, D314 (2 weeks after the second, third, and fourth vaccination respectively), and D356 (8 weeks after the fourth vaccination), as in Fig 4A.

TBA, which assesses the prevalence of a mosquito infection, and TRA were evaluated by SMFA (S2 Table, SMFA with Individual Test Serum at Each Time Point). Since TBA results are highly variable and dependent upon the infection challenge in the SMFA [28, 29], only TRA results, tested in replicate in 2 separate experiments, are presented in Fig 5A. The TRA, i.e., reduction in infection intensity, was measured with sera collected on Days 70, 134, 314, and 356: two weeks after the second and third vaccinations, and 2 and 8 weeks after the booster dose. With knowledge of the inherent variability in the SMFA, we consider >50% as a minimal threshold TRA likely to represent true biological activity; this is also reflected by the trend toward lower SD at higher TRA average values as displayed in Fig 5B. A majority of sera collected 2 weeks post the second and third vaccination in the Group 2 participants were below that threshold, whereas 2 weeks after the booster vaccination 9 out 11 Group 2 participants surpassed the 50% activity threshold. As the antibody levels declined over time, so did the TRA, as measured in SMFA with sera collected on Day 356, 8 weeks after the booster dose.

Using a linear GEE model to the log of the oocyst count ratio (test/control) from the SMFAs using the square root of the ELISA values on sera from Days 70, 134, and 314 sera, we find that anti-Pfs25 levels are highly associated with SMFA values; P-value <0.001. Using the same model, the estimated EC_50_ of serum anti-Pfs25 level from our GEE method is 57.2 μg/mL (95% CI, 44.7, 76.8 μg/mL).

### Antibody avidity

**Fig 6:**
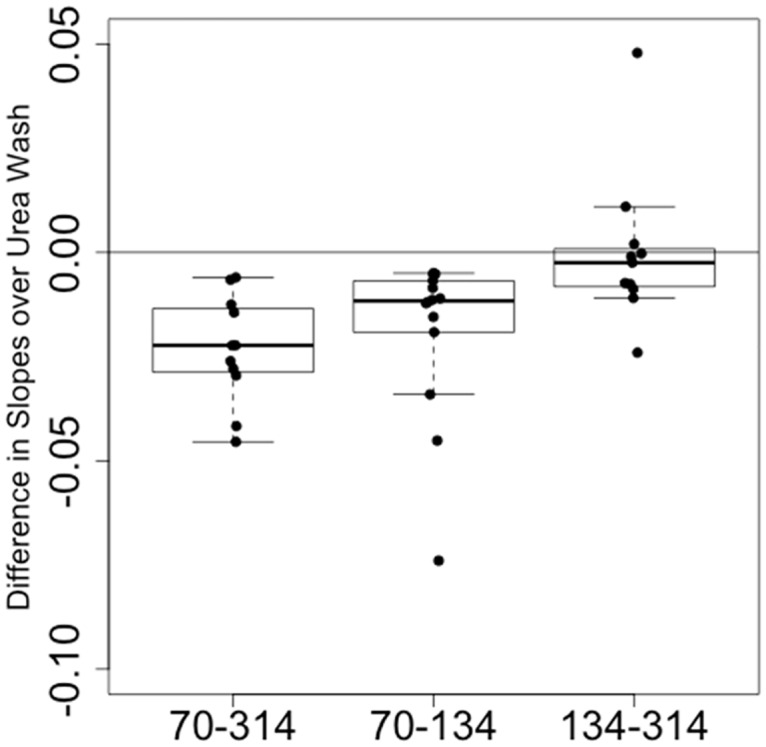
Paired comparison of Avidity Index in Sera after Subsequent Vaccinations.

To address whether the quality of the antibody improved over successive vaccinations, 16 sets of sera, collected on Day 70, 134, and 314 (2 weeks post the second, third, and the booster dose) from 16 individual participants in which 12 received the booster dose, were evaluated for antibody avidity by a urea-displacement ELISA. In this assay, ascending urea concentration in the wash buffer results in reduced antibody retention in the ELISA wells, and thus in decreasing OD_450_; as the avidity of the sample increases, the change, i.e., the slope of OD_450_ at ascending urea concentration approaches zero (0). As demonstrated in Fig 6 and S1 Fig (Slope of Change as Avidity Index of D70, D134, D314 Sera from Individual Participants), there was a significant increase in avidity from Day 70 to 134 and from Day 70 to 314 sera (both Wilcoxon signed-rank *P*-values <0.001); we did not find evidence of an increase from Days 134 to 314 (*P-value* = 0.431). We find some evidence that increased avidity is associated with increased TRA in Day 314 sera, after adjustment for Day 314 anti-Pfs25 ELISA titers, (*P-value* = 0.045). There was no evidence to support that this association also occurred in the day 134 and day 70 sera.

## Discussion

The challenges in TBV development and characteristics of an ideal TBV have recently been described [30, 31]. In order to break the cycle of malaria transmission, transmission blocking vaccines may require mass administration to a broad population, including infants, children, and women of reproductive age. Further, while herd immunity will result in indirect protection, the benefit to an individual receiving the vaccine is indirect and delayed. Thus the safety profile for a TBV must be highly favorable, and a formulation with less perceived risk is an advantage for rapid clinical development. The results in this study showed the Pfs25-EPA/Alhydrogel^®^ formulation to be well tolerated at all dose levels, with most local and systemic adverse events being mild in severity, no apparent increase in adverse events with successive vaccinations, and no participants withdrawn due to adverse events.

Immunogenicity was also demonstrated, with most participants having detectable antibody responses after two doses of vaccine, and all participants who received three doses of vaccine having detectable responses. Antibody levels were the highest after 4 vaccinations, administered to the highest dose group (47 μg Pfs25-EPA), with a geometric mean of 88 μg/mL obtained 2 weeks after the last dose. This level is not far from the antibody levels induced after vaccination with powerful adjuvants such as CPG 7909 [32]. Antibody levels declined rapidly, a finding that was not unexpected with the use of an aluminum adjuvant. In contrast to the vaccine-induced immunity against Pfs25H, the anti-EPA antibodies were detectable after the first vaccination, and their rate of decline was slower, possibly due to prior exposure to *Pseudomonas* bacteria, albeit anti-EPA antibodies were undetectable in pre-immune sera from participants.

The induced antibodies recognized native protein on parasite surface and were active in the SMFA, and the transmission reducing activities correlated with antibody titers measure by ELISA. We also found evidence of a weak association between TRA and avidity in Day 314 sera. However, we did not find a significant association between TRA and avidity in Day 70 and Day 134 sera, where TRAs of individual sera were low and highly variable. The EC_50_ was estimated to be 57.2 μg/ml (95% CI 44.7, 76.8). This compares to an EC_50_ of 85.6 μg/mL (95% CI 58.1–126.0) from serum obtained in a previous trial with Pfs25/ISA51 [14, 24], suggesting that the multi-dose Pfs25-EPA regimen may have increased antibody avidity and hence activity in the SMFA. However, it should be noted that the EC_50_ values from the two studies used different statistical models, since the EC_50_ value from the Pfs25/ISA51 trial was calculated from TRA data using purified IgGs primarily from a single volunteer [14], whereas the EC_50_ value from the Pfs25-EPA/Alhydrogel^®^ trial used the Day 70, 134, and 314 sera from Group 2 participants with various anti-Pfs25 titers. If the EC_50_ due to the antibodies from the single volunteer that dominated the first analysis [24] were atypical, this could have caused the apparent difference in EC_50_ between the two studies.

It is not known the level of transmission reducing or blocking activity required to have a meaningful impact in preventing malaria transmission in the field. The SMFA has been developed to investigate biologic impact of induced and natural transmission blocking antibody, but as a biologically complex assay involving feeding of laboratory-reared mosquitoes through a membrane, it is highly variable and difficult to consistently reproduce, especially when the blocking activity of the test samples are weak-to-moderate. Importantly, the TBA measurement is highly dependent on the control oocyst intensity which is difficult to reproduce; the TRA is much less dependent on that value [28, 29], and hence is the result presented in this paper. The assay reproducibility may be improved by using purified IgG; and the reproducibility is generally higher in feeding experiments where the test sample has high TRA (>80%) and the mean oocyst count in the assay control is in a moderate range of 8–26 [28, 29]. Analyzing the actual data of feeding experiments, where the mean oocyst counts in the assay control ranges from 19 to 113, we also found that the assay standard deviation (SD) between replicates was significantly lower at TRA values larger than 50% (Wilcoxon rank sum test comparing SD of SMFA with average TRA >50% to those <50%, P-value = 0.01). Thus, we selected 50% TRA as a threshold for likely reliable biological activity for this Phase 1 study. However, in the field oocyst counts in infected mosquitoes are typically low, in the range of 2–10 [33–35]. Whether this 50% TRA threshold assessed by the SMFA is sufficient to impact transmission in the field (i.e. to reduce the oocyst count in a mosquito from 2–10 to 0) is unknown. A Phase 1b trial of Pfs25-EPA/Alhydrogel^®^ is currently being conducted in Malian adults [ClinicalTrials.gov Identifier: NCT01867463], and field trials of transmission blocking vaccines will be needed to establish an association between results of the SMFA and results in vaccinated infected individuals.

It is likely that a more immunogenic vaccine than Pfs25-EPA/Alhydrogel^®^ will be needed to effectively interrupt malaria transmission. In particular, a formulation which induces consistently higher responses of longer duration may be required, with fewer vaccinations in the dosing regimen. Use of alternate conjugates and alternate adjuvants is being explored. Pfs25 may also be combined with other TBV or anti-disease vaccines such as RTS,S or equivalent which could permit use of more contemporary adjuvant formulations. However, the vaccine as currently formulated has shown an excellent safety profile and has induced biologically active levels of antibody. It is likely to be a very useful tool to elucidate the levels of antibody needed for biologic activity in malaria exposed populations, and to facilitate further development of assays to evaluate candidate transmission blocking vaccines. These results support further clinical testing in malaria-exposed populations.

## Supporting Information

S1 Fig Slope of Change as Avidity Index of D70, D134, D314 Sera from Individual Participants. (TIF)

S1 File Protocol: Open-Label Phase 1 Study in Malaria Naïve Adults of the Safety and immunogenicity of Pfs25-EPA/ALhydrogel, a Transmission Blocking Vaccine against *Plasmodium falciparum*. (PDF)

S2 File CONSORT 2010 checklist of information to include when reporting a randomized trial. (DOC)

S1 Table ELISA titer, IFA titer, and TRA of sera from selected volunteers. (DOCX)

S2 Table SMFA Results with Individual Test Serum at Each Time Point. (DOCX)
