# Supplementary figures and images for: Safety and Immunogenicity of Pfs25-EPA/Alhydrogel®, a Transmission Blocking Vaccine against Plasmodium falciparum: An Open Label Study in Malaria Naïve Adults
Source: PLoS One. 2016 Oct 17;11(10):e0163144. doi: 10.1371/journal.pone.0163144 (PMC5066979; doi:10.1371/journal.pone.0163144)

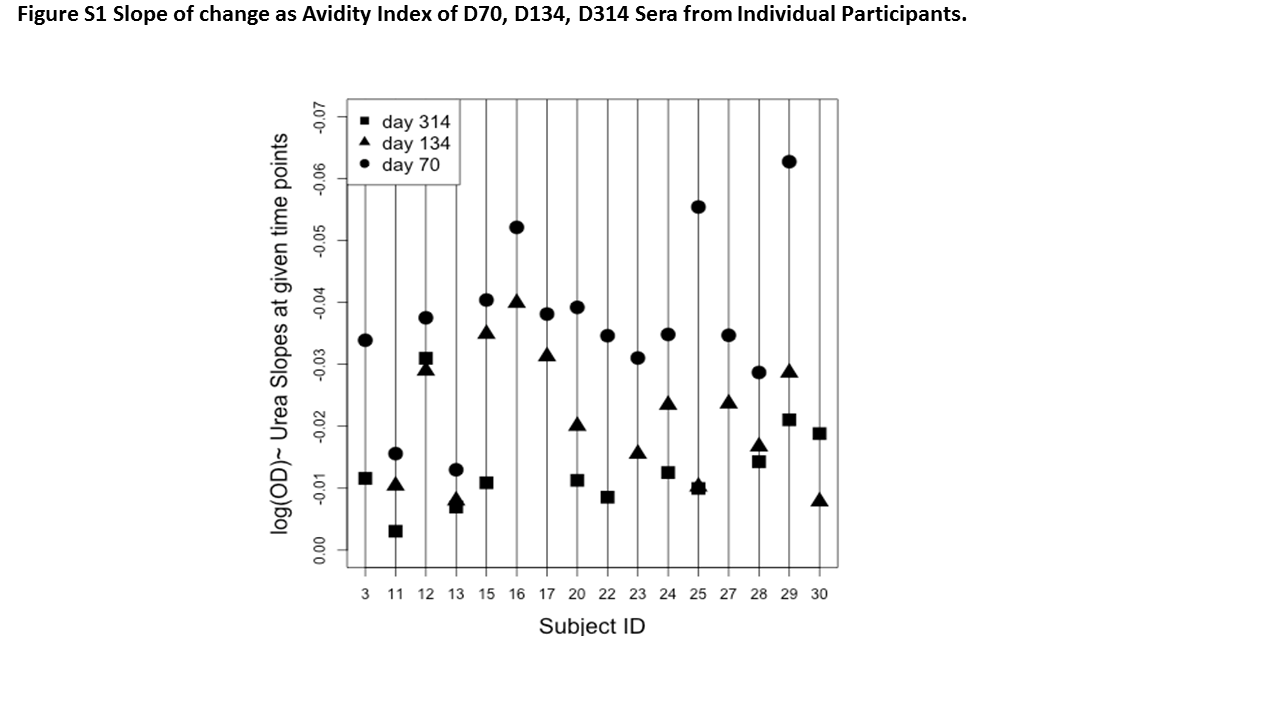

Supplement: S1 Fig — (TIF) [file pone.0163144.s001.tif]
