# Supplementary material for: Safety and Immunogenicity of Pfs25-EPA/Alhydrogel®, a Transmission Blocking Vaccine against Plasmodium falciparum: An Open Label Study in Malaria Naïve Adults
Source: PLoS One. 2016 Oct 17;11(10):e0163144. doi: 10.1371/journal.pone.0163144 (PMC5066979; doi:10.1371/journal.pone.0163144)
Supplement: S1 File — (PDF) [file pone.0163144.s002.pdf]

Open Label Phase 1 Study in Malaria Naïve Adults of the Safety and Immunogenicity of Pfs25-EPA/Alhydrogel®, a Transmission Blocking Vaccine Against *Plasmodium falciparum*

NIAID Protocol Number: 11-I-N237
Project Assurance: FWA #00005897
Multi-institution: Yes

CIR Protocol Number: 276
TBN IRB Protocol Number:
Project Assurance: FWA# 00000287 (Johns Hopkins Bloomberg School of Public Health)

Sponsored by:
National Institute of Allergy and Infectious Diseases (NIAID)
Laboratory of Malaria Immunology and Vaccinology (LMIV)
National Institutes of Health

IND Sponsor:
Regulatory Compliance and Human Subjects Protection Branch (RCHSPB)
National Institutes of Allergy and Infectious Diseases (NIAID)
IND: BB-IND # 14781

Funding Sponsor:
Program for Appropriate Technology in Health, Malaria Vaccine Initiative (PATH MVI)

Principal Investigators:
Kawsar Talaat, MD, Center for Immunization Research (CIR)
Patrick Duffy, LMIV/NIAID

Version: 6.0
9 January 2013

CONFIDENTIAL

Team Roster

Center for Immunization Research Principal Investigator:

Kawsar Talaat, MD
Center for Immunization Research (CIR)
Johns Hopkins School of Public Health
624 N. Broadway
Baltimore, MD 21205
410-614-4736

CIR Co-Investigators:

Anna P. Durbin, MD
Noreen A. Hynes, MD, MPH
Beulah P. Sabundayo, PharmD, MPH
Cecilia Tibery, PA-C, MS
Autumn Hentrich

NIAID Principal Investigator:

Patrick Duffy, MD
LMIV/NIAID/NIH
5640 Fishers Lane, Twinbrook I
Rockville, Maryland 20852
301-435-4605

NIAID Co- Investigator:

Yimin Wu, PhD
LMIV/NIAID/NIH

Biostatistician:

Michael P. Fay, PhD
NIAID/NIH
6700 A Rockledge Drive
Room 5133
Bethesda, Maryland 20892
301-451-5124

Participating Sites

**Clinical Trial Site**	Center for Immunization Research<br>Johns Hopkins School of Public Health<br>624 N. Broadway<br>Baltimore, MD 21205<br>410-614-4736
**Immunology Laboratories**	LMIV/NIAID/NIH<br>5640 Fishers Lane, Twinbrook I<br>Rockville, Maryland 20852<br>301-435-3405
	Laboratory of Malaria Vector Research (LMVR)<br>NIAID/NIH<br>12735 Twinbrook Parkway, Twinbrook III<br>Rockville, MD 20852 USA
	Pathogen & Vector Interaction Section (PVIS)<br>Entomology Department AFRIMS, THAILAND<br>315/6 Rajvithi, Rachathewee,<br>Bangkok 10400, Thailand
	Malaria Research and Training Center<br>MRTC/DEAP/FMPOS<br>Universite de Bamako<br>B.P. 1805, Bamako, Mali
	Institut de Recherche en Sciences de la Santé (IRSS)<br>Bobo-Dioulasso, Burkina Faso.<br>Institut de Recherche pour le Développement (IRD) - UMR MIVEGEC (UM1-UM2-CNRS 5290-IRD 224)<br>Montpellier, France
**Clinical Laboratory**	Quest Diagnostics<br>1901 Sulphur Spring Road<br>Baltimore, MD 21227

TABLE OF CONTENTS

TEAM ROSTER **2**

PARTICIPATING SITES **3**

TABLE OF CONTENTS **4**

LIST OF ABBREVIATIONS **7**

PROTOCOL SUMMARY **9**

PRÉCIS **11**

1 INTRODUCTION AND RATIONALE **12**

1.1 Malaria Epidemiology and Rationale for Vaccine 12
1.2 Description of Study Agent 12
1.2.1 Pfs25-EPA 12
1.2.2 Pfs25-EPA/Alhydrogel® 13
1.2.3 Storage and Shipping 13
1.3 Previous Human Experience 13
1.4 Clinical Development Plan 14

2 STUDY OBJECTIVES **14**

2.1 Primary Objective 14
2.2 Secondary Objectives 15
2.3 Exploratory Objectives 15

3 STUDY DESIGN **15**

3.1 Overall Design 15
3.2 Study Endpoints 17
3.2.1 Primary Endpoints 17
3.2.2 Secondary Endpoints 17
3.2.3 Exploratory Endpoints 17
3.3 Sample Size and Estimated Duration of Study 17

4 STUDY POPULATION **17**

4.1 Description of Population and Site 17
4.2 Recruitment 17
4.3 Inclusion Criteria 18
4.4 Exclusion Criteria 18

5 STUDY AGENT **20**

5.1 Vaccine 20
5.2 Vaccine Administration 20
5.3 Vaccine Storage 20
5.4 Vaccine Accountability 20

6 STUDY PROCEDURES **20**

6.1 SCREENING 20
6.2 ASSIGNMENT TO GROUPS 21
6.3 IMMUNIZATION PROCEDURE 21
6.4 CLINICAL MONITORING AND EVALUATION 21
6.5 LARGE VOLUME BLOOD DRAW 28
6.6 SYMPTOM MEMORY ENHANCEMENT CARD 28
6.7 PHOTOGRAPHS OF RASH OR INJECTION SITE REACTIONS 28
6.8 CONTRAINDICATIONS TO VACCINATION 28
6.9 INDICATIONS FOR DEFERRAL OF VACCINATION 29
6.10 SUBJECT WITHDRAWAL CRITERIA 29
6.11 REPLACEMENT OF SUBJECTS 30
6.12 TREATMENTS THAT COULD POTENTIALLY INTERFERE WITH VACCINE-INDUCED IMMUNITY 30
6.13 CLINICAL LABORATORY TESTING 31
6.14 IMMUNOLOGIC LABORATORY TESTING 31
6.14.1 Antibody Assay (ELISA) 31
6.14.2 Standard Membrane Feeding Assay (SMFA) 31
6.14.3 B-Cell and T-Cell Assays 32

7 USE, STORAGE, AND TRACKING OF SPECIMENS AND DATA 32

8 RETENTION OF SPECIMENS FOR FUTURE USE 33

9 SAFETY AND ADVERSE EVENT REPORTING 33

9.1 DEFINITIONS 33
9.2 PREEXISTING CONDITIONS, CONDITIONS, WORSENING OF PRE-EXISTING CONDITION 35
9.3 ASSESSMENT OF ADVERSE EVENTS 35
9.3.1 Adverse Event Definitions 36
9.3.2 Determination of Severity 36
9.3.3 Association with Receipt of the Study Vaccine 37
9.4 REPORTING 37
9.4.1 Reporting to IND Sponsor 38
9.4.2 Reporting to the IRBs 39
9.4.3 Reporting to Malaria Vaccine Initiative 39
9.4.4 Reporting to the Safety Monitoring Committee 40
9.5 HALTING RULES 40

10 CLINICAL MONITORING 41

10.1 SITE MONITORING PLAN 41
10.2 SAFETY MONITORING PLAN 41
10.2.1 Safety Monitoring Committee (SMC) 41

11 STATISTICAL CONSIDERATIONS 42

11.1 DESCRIPTION OF STATISTICAL METHODS 42
11.2 PRIMARY OBJECTIVE 42
11.3 SECONDARY OBJECTIVE 42
11.3.1 Exploratory Objectives 43
11.4 SAMPLE SIZE AND POWER CALCULATIONS 43
11.5 RANDOMIZATION 44

12 HUMAN SUBJECT PROTECTIONS AND ETHICAL OBLIGATIONS 44

12.1 INSTITUTIONAL REVIEW BOARD 44
12.2 INFORMED CONSENT PROCESS 44
12.3 JUSTIFICATION FOR EXCLUSION OF CHILDREN 45
12.4 SUBJECT CONFIDENTIALITY 45
12.5 RISKS 45
12.5.1 Venipuncture 45
12.5.2 Immunization 45
12.6 BENEFITS 46
12.7 COMPENSATION 46

13 DATA HANDLING AND RECORD KEEPING 46

13.1 SOURCE DOCUMENTATION 46
13.2 RETENTION OF STUDY RECORDS 47
13.3 PROTOCOL REVISIONS 47

REFERENCES 48

APPENDIX A: MALARIA COMPREHENSION EXAM 50

APPENDIX B: SCHEDULE OF PROCEDURES/EVALUATIONS 55

APPENDIX C: TOXICITY TABLE 61

TABLE OF TABLES

Table 1 Phase 1 Study of Pfs25-EPA Conjugates in US Healthy Adults 15
Table 2 Vaccination and Dose Escalation Schedule* 16
Table 3 Solicited Adverse Events 36
Table 4 Assessment of Relationship 37

List of Abbreviations

AE	adverse event/adverse experience
ALT	alanine transaminase
CBC	complete blood count
CFR	Code of Federal Regulations
cGMP	Current Good Manufacturing Practices
CIR	Center for Immunization Research (Johns Hopkins Univ.)
CRF	case report form
CRIMSON	Clinical Research Information Management System of the NIAID
DHHS	Department of Health and Human Services
ELISA	enzyme-linked immunosorbent assay
ELISPOT	enzyme-linked immunosorbent spot assay
EPA	ExoProtein A
FDA	Food and Drug Administration
FWA	Federal Wide Assurance
GCP	Good Clinical Practice
HBsAg	hepatitis B surface antigen
HCV	hepatitis C virus
HIV	human immunodeficiency virus
IB	Investigator's Brochure
IM	Intramuscular
IND	Investigational New Drug
IRB	Institutional Review Board
LMIV	Laboratory of Malaria Immunology and Vaccinology (of NIAID)
LMVR	Laboratory of Malaria Vector Research
MVI	Malaria Vaccine Initiative
N	number (typically refers to subjects or participants)
NIAID	National Institute of Allergy and Infectious Diseases, NIH
NIH	National Institutes of Health
OHRP	Office for Human Research Protections
OHSR	Office of Human Subjects Research
PATH	Program for Appropriate Technology in Health
Pfs25	Surface antigen of ookinetes in the mosquito stage of *Plasmodium falciparum*
PI	Principal Investigator
RCHSPB	Regulatory Compliance and Human Subjects Protection Branch
SAE	serious adverse event/serious adverse experience
SMFA	Standard membrane feeding assay
SOP	Standard Operating Procedure
TBA	Transmission blocking assay
TBV	Transmission blocking vaccine
WBC	white blood cell
WHO	World Health Organization

List of Abbreviations

WRAIR	Walter Reed Army Institute of Research
β-hCG	human choriogonadotropin

PROTOCOL SUMMARY

**Full Title:**	Open Label Phase 1 Study in Malaria Naïve Adults of the Safety and Immunogenicity of Pfs25-EPA/Alhydrogel®, a Transmission Blocking Vaccine Against *Plasmodium falciparum*
**Short Title:**	Phase 1 Study of Pfs25-EPA/Alhydrogel®
**Clinical Phase:**	1
**IND Sponsor:**	RCHSPB
**Conducted by:**	Center for Immunization Research
**Principal Investigators:**	Kawsar Talaat, MD (Johns Hopkins School of Public Health)<br>Patrick Duffy, MD (LMIV/NIAID)
**Sample Size:**	N= 30
**Accrual Ceiling:**	100
**Study Population:**	Malaria-naïve US adults
**Accrual Period:**	Approximately September 2011
**Study Design:**	Dose escalating, open label, Phase 1 clinical trial.<br>1. Group 1a: n=5, to receive 8 μg Pfs25-EPA/Alhydrogel® on D0, D56 +/- D300<br>2. Group 1b: n=5, to receive 16 μg Pfs25-EPA/Alhydrogel® on D0, D56 +/- D300<br>3. Group 2: n=20, to receive 47 μg Pfs25-EPA/Alhydrogel® on D0, D56, D120 and D300<br><br>Enrollment will be staggered for additional safety
**Study Duration:**	Start Date: Approximately September 2011<br>End Date: Approximately September 2013<br>Study participants will be enrolled for a total of 60 or 96 weeks, depending on vaccination schedule (12 months follow-up after the last vaccination)
**Study Agent/ Intervention Description:**	Pfs25-EPA: Recombinant Pfs25 is expressed in *Pichia pastoris*. Recombinant EPA, a mutant, non-toxic protein corresponding to sequence of ExoProtein A of *Pseudomonas aeruginosa,* is expressed in *E. coli*. Pfs25

and EPA are chemically crosslinked to form Pfs25-EPA conjugate. All recombinant proteins and the conjugate are manufactured and characterized in compliance with cGMP. Alhydrogel®, an adjuvant used in multiple licensed vaccines, is manufactured by Brenntag Biosector (Brenntag, Denmark).

Primary Objective: To assess safety and reactogenicity of Pfs25-EPA/Alhydrogel® in malaria-naïve adults

Secondary Objectives: To determine the antibody response to the Pfs25 protein vaccines as measured by ELISA and transmission blocking assays and the effect on antibody responses of a third dose at 4 months and a booster dose at 10 months

Exploratory Objectives: T and B cell responses to vaccination

Endpoints:

1. Incidence of local and systemic adverse events
2. Antibody levels elicited by Pfs25-EPA, as measured by ELISA
3. Transmission blocking activity (TBA) of induced antibody, and correlation of TBA with antibody levels
4. T cell responses to Pfs25 as measured by cytokine levels and ELISPOT assays
5. The number and proportion of Pfs25 specific memory B cells as measured by flow cytometry and ELISPOT assay

Précis

A vaccine to interrupt malaria transmission would be a valuable tool for local elimination or eradication of this disease. Pfs25, a surface antigen of ookinetes in the mosquito stage of *P. falciparum*, is a lead candidate for a malaria transmission blocking vaccine. Recombinant Pfs25 has been conjugated to *Pseudomonas aeruginosa* ExoProtein A (EPA), and adjuvanted with Alhydrogel®. This open label, dose escalating Phase 1 study in malaria naïve adults, conducted at Johns Hopkins Bloomberg School of Public Health Center for Immunization Research (CIR) in Baltimore, Maryland, will determine initial safety and immunogenicity of the vaccine given on a 2 dose and 3 dose schedule. Thirty (30) volunteers will be enrolled, with 5 receiving the lowest dose (8 µg of conjugated Pfs25), 5 receiving the middle dose (16 µg of conjugated Pfs25), and 20 receiving the high dose (47 µg of conjugated Pfs25). The low dose groups will receive 2 doses of vaccine and the high dose group will receive 4 doses of vaccine, on a 0, 2 month (low dose) or 0, 2, 4 and 10 month (high dose) vaccination schedule. In addition, extra-ordinary responders to the low dose of vaccine (as measured by antibody level) will be offered a booster dose at 10 months. Volunteers will be followed for 12 months following the last vaccination. Safety outcomes will be local and systemic adverse events (AEs). Immunogenicity outcomes will be antibody responses as measured by ELISA, transmission blocking in a standard membrane feeding assay, and B and T cell responses.

1 Introduction and Rationale

1.1 Malaria Epidemiology and Rationale for Vaccine

According to the World Health Organization (WHO), global control efforts have resulted in a reduction in the number of deaths since 2000; however 781,000 people are estimated to have died due to malaria in 2009 [World malaria report 2010]. Morbidity and mortality caused by malaria also has significant direct and indirect costs on the economic development of endemic countries [Sachs J, Malaney P 2002]. These factors, as well as growing drug resistance of the parasite, widespread resistance of mosquitoes to insecticide, and increased human travel necessitate new approaches to malaria control and eradication. A vaccine that interrupts *P. falciparum* transmission would be a valuable resource in the fight against this disease [Greenwood B, Targett D 2009; Birkett AJ 2010]. Transmission blocking vaccines (TBVs) aim to induce antibody in the human host that is taken up with the blood meal and blocks parasite development in the mosquito, thereby halting transmission to another human host.

Pfs25, a surface antigen of ookinetes in the mosquito stage of *P. falciparum*, has long been a lead candidate for developing a malaria TBV [Kaslow DC 2002]. However, soluble recombinant Pfs25 is poorly immunogenic. The Laboratory of Malaria Immunology and Vaccinology (LMIV) and collaborators have chemically conjugated Pfs25 to the Outer Membrane Protein Complex of *Neisseria meningitidis*, to Exo Protein A (EPA), a mutant and detoxified protein from *Pseudomonas aeruginosa*, or to Pfs25 itself to form multimeric Pfs25. These conjugates induced sustained, significantly higher antibody responses in mice, rabbits, and rhesus monkeys than un-conjugated Pfs25 [Wu et al 2006; Kubler-Kielb et al 2007; Qian et al 2007]. Recombinant EPA is not a component of any licensed vaccines, but has been extensively studied as a component of a conjugated typhoid vaccine, in a similar dose range as that which will be used in this study (22 µg vs. 56 µg) [Lin et al 2001]. Multiple batches of Pfs25-EPA conjugate were produced and biochemical characteristics of these batches were comparable. Anti-conjugated Pfs25-EPA antibodies induced in mice and guinea pigs were associated with strong blocking of parasite development in mosquitoes in an ex vivo standard membrane feeding assay (see the Investigators Brochure). Aluminum based adjuvants are widely used, both to stabilize protein vaccines and to enhance immune responses. Pfs25-EPA will be formulated with Alhydrogel®.

This dose-escalating first in human Phase 1 study will provide data on reactogenicity and immunogenicity of adjuvanted Pfs25-EPA conjugate vaccines given on 2 different dosing schedules.

1.2 Description of Study Agent

1.2.1 Pfs25-EPA

The cGMP lots PpPfs25H Lot# WRAIR1390 and EcEPA Lot# WRAIR1365, both manufactured at Walter Reed Bioproduction facility (Silver Spring, Maryland) in cGMP compliance, were used

to manufacture the conjugate vaccine. PpPfs25H is a *Pichia*-expressed hexa-His tagged recombinant Pfs25 with a molecular mass of 20,437 Daltons. EcEPA is an *E. coli*-expressed recombinant protein with molecular mass of 66,975 Daltons. The Pfs25-EPA conjugate was produced by reaction between thiolated PpPfs25 and maleimide-activated EcEPA, followed by purification using size-exclusion chromatography.

The cGMP Pfs25-EPA conjugate Lot# WRAIR1634 was manufactured at Walter Reed Bioproduction facility in cGMP compliance in May 2010.

1.2.2 Pfs25-EPA/Alhydrogel®

Alhydrogel®, an aluminum hydroxide gel (Brenntag, Denmark), has been extensively used as an adjuvant in many licensed human vaccines. Alhydrogel® is supplied as a sterile product in water without preservatives. Pfs25-EPA/Alhydrogel® WRAIR Lot #1668 was manufactured and filled as single-use vials at Walter Reed Bioproduction facility in cGMP compliance in October 2010. Each vial contains 78 µg/mL conjugated Pfs25, 93 µg/mL conjugated EPA and 1600 µg/mL Alhydrogel® in a volume of 0.8 mL. The vial label reads: 78 µg/mL Conjugated Pfs25 on Alhydrogel®.

An injection volume of 0.1 mL will deliver 8 µg conjugated Pfs25, 9 µg conjugated EPA, and 160 µg Alhydrogel®; 0.2 mL will deliver 16 µg conjugated Pfs25, 19 µg conjugated EPA, and 320 µg Alhydrogel®; and a volume of 0.6 mL will deliver 47 µg conjugated Pfs25, 56 µg conjugated EPA, and 960 µg Alhydrogel®.

1.2.3 Storage and Shipping

Pfs25-EPA/Alhydrogel® should be stored at 2-8°C. Pfs25-EPA/Alhydrogel® will be transported at temperature-controlled conditions, as per LMIV's Standard Operating Procedure (SOP). Temperature data loggers will accompany the vaccines at all times during transport to ensure temperature limits have not been violated.

1.3 Previous Human Experience

This Phase 1 study is the first time that this conjugated vaccine will be given to humans. The components of the vaccine (unconjugated antigen and adjuvants) have been evaluated in prior clinical trials described below. Details on adverse events (AEs) and immunogenicity results of these trials are described in the Investigator's Brochure.

Pfs25 formulated with the water-in-oil emulsion Montanide ISA51 was evaluated in a dose escalating Phase 1 trial in malaria naïve adults, together with the *Plasmodium vivax* homologous antigen Pvs25 [Wu et al 2008]. Vaccinations were halted due to local and systemic reactogenicity, including 2 cases of erythema nodosum in subjects who received 1 dose of 20 µg Pvs25/ISA51, and 2 leukemoid reactions (one in the Pfs25 5 µg group which occurred 14 days after second vaccination, and one in the Pvs25 20 µg group which occurred 120 days after first vaccination). Ten subjects received 1 dose of 5 µg Pfs25/ISA51; 2 subjects were withdrawn due to severe local reactions and 3 others were withdrawn for unrelated reasons.

Of the 5 subjects who received a second dose of vaccine, all had detectable antibody responses, with 1 subject's responses peaking at 7322 ELISA units. Serum from this subject resulted in >90% reduction of oocyst counts in a transmission blocking assay; serum from 3 of the other 4 responders was also tested and showed moderate transmission blocking activity (18-47% reduction in oocysts), with transmission blocking activity correlating with anti-Pfs25 antibody levels.

Clinical development was halted due to the unfavorable safety profile of the Montanide ISA51 formulation. While some degree of local reactogenicity was expected with this water-in-oil formulation, the systemic events which occurred were not predicted by animal toxicity studies or previous human experience with these antigens or with this adjuvant.

The same Pvs25 antigen formulated with Alhydrogel® had previously been well tolerated, if poorly immunogenic [Malkin et al 2005]. A similar recombinant Pfs25 (expressed in *Saccharomyces* but with the same amino acid sequence), was generally well tolerated when given with alum and in a prime-boost sequence, although 1 subject had an atypical contralateral hypersensitivity reaction thought to be due to free antigen dissociated from the alum [Kaslow 2002]. Pfs25 is structurally similar to Pvs25, but the 2 proteins have only ~45% sequence homology. Given all of the above, the unexpected systemic events which occurred with the Pvs25/ISA51 formulation are most likely due to that specific antigen-adjuvant combination.

Alhydrogel® is a licensed, widely used adjuvant, thought to enhance immune responses through a depot effect and by stimulation of the Nalp3 inflammasome [Eisenbarth et al 2008]. Local AEs such as pain, swelling and erythema can occur, as well as subcutaneous nodules believed to be granulomatous reactions to aluminum.

1.4 Clinical Development Plan

This vaccine is intended to interrupt malaria transmission by blocking infection in mosquitoes. The biologic activity of antibody induced by vaccination is demonstrated by a membrane feeding assay, as described in Section 6.14.2. If safety and immunogenicity are demonstrated in this study (Phase 1a) a Phase 1 study in malaria exposed adults (Phase 1b) is planned. In that study direct feeding assays will be conducted in parallel with membrane feeding assays. If transmission blocking in vaccinated individuals is demonstrated by direct feeds this would be an important step towards establishing likely benefit and efficacy of TBVs.

2 Study Objectives

2.1 Primary Objective

The primary objective of this study is to assess safety and reactogenicity of Pfs25-EPA/Alhydrogel® in malaria-naïve adults.

2.2 Secondary Objectives

The secondary objectives of this study are to determine the antibody response to the Pfs25 protein vaccines as measured by ELISA and transmission blocking assays, and the effect on antibody responses of a third and fourth (booster) doses at 4 and 10 months, or 2 doses 2 months apart and a booster 10 months later. In addition, plasma obtained during the study will be used to create a high-titer antibody standard reference reagent for research assays.

2.3 Exploratory Objectives

Exploratory objectives of this study include the following:

- To determine T and B cell responses to the Pfs25 protein vaccine.

3 Study Design

3.1 Overall Design

Objectives of this open label Phase 1 study are to evaluate safety, immunogenicity, and transmission blocking activity of Pfs25-EPA conjugates formulated on Alhydrogel®. The study will enroll 30 volunteers as shown in **Table 1**. The volunteers will be followed for 12 months after the last immunization.

Table 1 Phase 1 Study of Pfs25-EPA Conjugates in US Healthy Adults

Group	N	Vaccine	Schedule (month)
1a	5	8 µg Pfs25-EPA/Alhydrogel®	0, 2, optional 10
1b	5	16 µg Pfs25-EPA/Alhydrogel®	0, 2, optional 10
2	20	47 µg Pfs25-EPA/Alhydrogel®	0, 2, 4, 10

Groups 1a and 1b are sized for safety rather than immunogenicity, as the higher dose is expected to be necessary for an adequate immune response. Participants in Group 2 will receive 4 doses of vaccine to allow estimation of the effect of the additional dose of vaccine on immunogenicity. A fourth dose was added to explore if a booster dose will increase antibody responses, as preliminary results suggest that antibody responses increase with each of the first 3 doses. In addition, subjects of Group 1a and Group 1b who were exceptionally high (>500 EU) anti-Pfs25 antibody producers as measured by ELISA assay after the second dose will be offered the opportunity to receive a booster dose at 10-11 months. The purpose of this is to obtain high-titer antibodies and evaluate whether transmission blocking activity correlates with the antibody titers. Table 2 shows the vaccination and dose escalation schedule for the 2 groups; first vaccinations within dose groups are staggered for assessment of safety of the vaccine in a small number of subjects in each dose group prior to further enrollments.

Table 2 Vaccination and Dose Escalation Schedule*

Week	Vaccination 1	Vaccination 2	Vaccination 3	Vaccination 4
0	**n=2:**<br>8 ug Pfs25-EPA/ Alhydrogel®<br>(Group 1a)			
1	**n=3:**<br>8 ug Pfs25-EPA/ Alhydrogel®<br>(Group 1a)			
2	**n=5:**<br>16 ug Pfs25-EPA/ Alhydrogel®<br>(Group 1b)			
3	**n=5**:<br>47 ug Pfs25-EPA/ Alhydrogel®<br>(Group 2)			
4	**n=15**:<br>47 ug Pfs25-EPA/ Alhydrogel®<br>(Group 2)			
8		**n=5:**<br>8 ug Pfs25-EPA/ Alhydrogel®<br>(Group 1a)		
10		**n=5:**<br>16 ug Pfs25-EPA/ Alhydrogel®<br>(Group 1b)		
12		**n=20**:<br>47 ug Pfs25-EPA/ Alhydrogel®<br>(Group 2)		
20			**n=20**:<br>47 ug Pfs25-EPA/ Alhydrogel®<br>(Group 2)	
26				**n=20:**<br>47 ug Pfs25-EPA/ Alhydrogel®<br>(Group 2 +/- Group 1 high responders)

*Schedule is approximate; a minimum of 5 days will separate the last subjects vaccinated at the previous dose group from the first vaccinated in the subsequent dose group.

3.2 Study Endpoints

3.2.1 Primary Endpoints

The incidence and severity of systemic and local AEs

3.2.2 Secondary Endpoints

1. Antibody levels elicited by Pfs25-EPA, as measured by ELISA
2. Transmission blocking activity (TBA) of induced antibody as measured in standard membrane feeding assays (SMFA), and correlation of TBA with antibody levels

3.2.3 Exploratory Endpoints

1. T-cell responses to Pfs25 as measured by cytokine levels and ELISPOT assays
2. The number and proportion of Pfs25 specific memory B cells will be measured by flow cytometry and ELISPOT assays

3.3 Sample Size and Estimated Duration of Study

A total of 30 subjects will be vaccinated in this trial. Each subject will be monitored actively for approximately 12 months after the last injection, for a total of 60 or 100 weeks, depending on the vaccination schedule. Up to 100 volunteers will be screened to accommodate possible screening failures.

4 Study Population

4.1 Description of Population and Site

The study will be conducted at the Johns Hopkins Bloomberg School of Public Health, Center for Immunization Research (CIR) in Baltimore, Maryland. CIR has successfully conducted 4 previous Phase 1 studies of malaria vaccine candidates in collaboration with LMIV. Malaria-naïve volunteers will be recruited from the surrounding community through a screening protocol as described in **Section 4.2**.

4.2 Recruitment

Healthy adult male and non-pregnant female volunteers will be recruited from a variety of sources including those previously screened or enrolled in other vaccine trials at the CIR, or by the use of an Institutional Review Board (IRB)-approved screening protocol and study-specific print or media advertising. After an initial phone screen (using an IRB approved Phone Screen/ Initial Contact form) by clinic staff consisting of providing background information of the trial and a review of basic inclusion and exclusion criteria, a screening visit will be scheduled.

During the screening process, which may require more than one visit, the volunteer will read the consent form, be encouraged to ask questions, and then complete a written comprehension evaluation questionnaire (Appendix A). The questionnaire is used to identify the areas of the

study and consent that the volunteer may not fully understand. The person administering consent reviews the answers with the volunteer. If the volunteer gets a question wrong, the person administering the consent reviews the portion of the consent form that relates to that particular question with the volunteer. The volunteer may either sign the consent form during the screening visit, or return after further consideration.

4.3 Inclusion Criteria

All of the following criteria must be fulfilled for a volunteer to participate in this trial:

1. Age between 18 and 50 years.
2. Good general health as a result of review of medical history and/or clinical testing at the time of screening.
3. Available for the duration of the trial.
4. Willingness to participate in the study as evidenced by signing the informed consent document.
5. If female: subject is willing to use reliable contraception methods for the period of at least 1 month (2 months for oral contraceptive pills) prior to first vaccination to 3 months after last vaccination. Reliable methods of birth control include: pharmacologic contraceptives including oral, parenteral, and transcutaneous delivery; condoms with spermicide; diaphragm with spermicide; surgical sterilization; vaginal ring; transdermal patch; intrauterine device; abstinence; and post-menopause.

4.4 Exclusion Criteria

A volunteer will be excluded from participating in this trial if any one of the following criteria is fulfilled:

1. Pregnancy as determined by a positive urine or serum human choriogonadotropin (β-hCG) test at any point during the study (if female).
2. Currently is lactating and breast-feeding (if female).
3. Behavioral, cognitive, or psychiatric disease that in the opinion of the investigator affects the ability of the participant to understand and cooperate with the study protocol.
4. Neutropenia as defined by an absolute neutrophil count $<1500/mm^3$.
5. Alanine transaminase (ALT) level above the laboratory-defined upper limit of normal.
6. Evidence of clinically significant neurologic, cardiac, pulmonary, hepatic, endocrine, rheumatologic, autoimmune, hematological, or renal disease by history, physical examination, and/or laboratory studies including urinalysis.
7. Other condition that in the opinion of the investigator would jeopardize the safety or rights of a participant participating in the trial or would render the subject unable to comply with the protocol.

8. History of receiving any investigational product within the past 30 days.
9. Receipt of antimalarial prophylaxis during the past 12 months, or planned travel to a destination which would require malaria prophylaxis during the period of participation.
10. Prior malaria infection by history.
11. Participant has had medical, occupational, or family problems as a result of alcohol or illicit drug use during the past 12 months.
12. History of a severe allergic reaction or anaphylaxis.
13. Severe asthma. This will be defined as:
    - Asthma that is unstable or required emergent care, urgent care, hospitalization or intubation during the past 2 years, or that requires the use of oral or parenteral corticosteroids.
    - Clinically significant reactive airway disease that does not respond to bronchodilators.
14. Positive ELISA and confirmatory Western blot tests for HIV-1.
15. Positive ELISA and confirmatory tests for hepatitis C virus (HCV).
16. Positive hepatitis B surface antigen (HBsAg) by ELISA.
17. Pre-existing autoimmune or antibody-mediated diseases including but not limited to: systemic lupus erythematosus, rheumatoid arthritis, multiple sclerosis, Sjögren's syndrome, or autoimmune thrombocytopenia.
18. Known immunodeficiency syndrome.
19. Use of chronic (≥14 days) oral or intravenous corticosteroids (excluding topical or nasal) at immunosuppressive doses (i.e. prednisone >10 mg/ day) or immunosuppressive drugs within 30 days of starting this study.
20. Receipt of a live vaccine within past 4 weeks or a killed vaccine within past 2 weeks prior to entry into the study.
21. History of a surgical splenectomy.
22. Receipt of blood products within the past 6 months.
23. Previous receipt of an investigational malaria vaccine.
24. Refusal to allow storage of samples for future research.
25. Any medical, psychiatric, social, or occupational condition or other responsibility that, in the judgment of the Principal Investigator (PI), would interfere with the evaluation of study objectives.

5 Study Agent

5.1 Vaccine

The Pfs25-EPA/Alhydrogel® vaccine will be supplied to the study site pharmacist by the Pilot Bioproduction Facility, Walter Reed Army Institute of Research, Silver Spring, MD where the materials were formulated and packaged. Vaccines will be labeled for investigational use only.

5.2 Vaccine Administration

The Pfs25-EPA/Alhydrogel® vaccine will be provided as a single-use vial. A 0.1 mL volume is administered for delivery of 8 µg conjugated Pfs25, a 0.2 mL volume is administered for delivery of 16 µg conjugated Pfs25, and a 0.6 mL volume is administered for delivery of 47 µg conjugated Pfs25. The vaccine can be drawn up into the syringe up to 5 hours prior to administration and will be mixed by hand before injection to ensure resuspension.

5.3 Vaccine Storage

Pfs25-EPA/Alhydrogel® vaccines are stored at 2-8 °C and may NOT be frozen at any time. Refrigerator temperature will be continuously monitored using an alarm system.

5.4 Vaccine Accountability

After administration of a vaccine dose, the single-dose vial will be returned to the storage facility at the test site, and vials will be accounted for and stored until monitoring by the IND Sponsor. The used vials may then be disposed of according to site standard operating procedures after monitoring has been completed.

The trial site pharmacist is responsible for maintaining an accurate inventory and accountability record of vaccine supplies for this study. Partially used vials may not be administered to other subjects.

6 Study Procedures

6.1 Screening

The purpose of the screening visit (as described in Section 4.2) is to determine volunteer eligibility for study participation. Subjects who are diagnosed with a medical condition during the screening process (e.g., test positive for hepatitis B, hepatitis C, or HIV) will be notified and referred for medical care when possible according to Maryland reporting requirements.

The following screening procedures and evaluations for this study must be completed within 60 days prior to first vaccination.

- Explain the study and Informed Consent to the subject.
- Ensure the subject has successfully completed the Informed Consent Comprehensive Exam, has signed the Informed Consent and receives a signed copy of the Informed Consent

- Ensure that HIV pre-test counseling has been performed and ensure that the subject has agreed to HIV testing (required by Maryland state law).
- Elicit a complete medical history, including menstrual and contraceptive history and/or history of surgical sterility for females.
- Pregnancy prevention counseling will be performed.
- Administer a complete physical examination, including vital signs (height, weight, blood pressure, temperature, respirations, and pulse).
- Obtain a signed medical release of information form from the patient.
- Obtain approximately 20 mL of blood for complete blood count (CBC) with differential and platelet count, ALT, creatinine, hepatitis B surface antigen, hepatitis C antibody, HIV antibody.
- Obtain urine for pregnancy testing and dipstick testing for protein and blood.

6.2 Assignment to Groups

Subjects in Group 1a will be enrolled first, followed by Group 1b, and Group 2. (Note: subjects in Group 2 were initially randomly assigned to a 2 or 3 dose vaccination schedule (Group 2a or 2b); the protocol was subsequently amended (Version 3) such that all subjects in Group 2 receive 3 doses of vaccine). The protocol was again amended (Version 4) so that the subjects in Group 2 can receive an additional booster 6-8 months later if they received at least 2 previous doses of the vaccine, and so that subjects in Group 1 can also receive the booster 8-9 months later if they had an exceptionally high antibody titer response to the first 2 doses (>500 EU).

6.3 Immunization Procedure

Vaccines will be administered IM in the deltoid muscle. Arms may be alternated with successive vaccinations.

6.4 Clinical Monitoring and Evaluation

The study schedule and approximate amounts of blood drawn are summarized in Appendix B.

<u>All Groups</u>

<u>Study Day 0 (Day of First Vaccination)</u>

1. Verify that Informed Consent was obtained.
2. Ensure that all inclusion/exclusion criteria are met.
3. Ensure that CBC, ALT, and creatinine measurements from screening tests are within protocol-defined limits (Section 4.4 Exclusion Criteria) before vaccinating.
4. Perform interim history and physical examination, focusing on any acute complaints.
5. Obtain blood for CBC with differential and platelet count, ALT, creatinine, anti-Pfs25 antibody ELISA, SMFA, and B and T cell studies.
6. Obtain a urine sample for dipstick testing and ensure that protein and blood are no more than

trace positive before vaccinating. Should a female subject have more than trace blood in her urine on Day 0, she will not be excluded from vaccination if she is currently menstruating.

7. For females, obtain a urine or serum sample for β-hCG testing. Ensure that the test is negative before vaccinating; a positive test will exclude the subject from the trial. Pregnancy prevention counseling will be performed.
8. Record vital signs (blood pressure, temperature, heart rate, and respiratory rate).
9. Administer the vaccine.
10. Observe for at least 30 minutes after vaccination to evaluate for immediate adverse reactions.
11. Education by study staff during 30-minute post-immunization wait period, describing proper use of digital thermometer, injection-site reaction measurement, and malaria vaccine side-effect memory enhancement card. Study staff will also discuss signs and symptoms of potential AEs, and indications for use of antipyretics (acetaminophen or ibuprofen) if fever, headache, or malaise occurs.

Study Day 3 ±1 Day

1. Perform basic history and focused physical examination (including injection site), emphasizing examination of any acute complaints.
2. Review memory enhancement card.
3. Record vital signs.
4. Obtain blood for CBC with differential and platelet count, ALT, and creatinine.

Study Day 7 ±1 Day

1. Perform basic history and focused physical examination (including injection site), emphasizing examination of any acute complaints.
2. Record vital signs.
3. Obtain blood for CBC with differential and platelet count.
4. Obtain urine for dipstick testing.
5. Review Days 0-6 memory enhancement card.

Study Day 14 ±2 Days

1. Perform basic history and focused physical examination (including injection site), emphasizing examination of any acute complaints.
2. Record vital signs.
3. Obtain urine for dipstick testing. Obtain urine or serum for β-hCG testing.
4. Obtain blood for CBC with differential and platelet count, ALT, creatinine, anti-Pfs25 antibody ELISA.
5. Review pregnancy prevention counseling.

Study Day 28 ±3 Days

1. Perform directed history and assessment of any physical complaint.
2. Record vital signs.
3. Obtain urine for dipstick testing.
4. Review pregnancy prevention counseling.

Study Day 56 +14 (Day of Second Vaccination)

1. Perform basic history and focused physical examination, emphasizing examination of any acute complaints.
2. Review criteria for vaccination.
3. Obtain blood for CBC with differential and platelet count, ALT, creatinine, anti-Pfs25 antibody ELISA, and B and T cell studies.
4. For females, obtain a urine or serum sample for β-hCG testing. Ensure that the test is negative before vaccinating; a positive test will exclude the subject from the trial. Pregnancy prevention counseling will be reviewed.
5. Obtain a urine sample for dipstick testing and ensure that protein and blood are no more than trace positive before vaccinating. Should a female subject be menstruating on Day 56, she will not be excluded from vaccination if she has more than trace blood in her urine.
6. Record vital signs (blood pressure, temperature, heart rate, and respiratory rate).
7. Administer the vaccine.
8. Observe for at least 30 minutes after vaccination to evaluate for immediate adverse reactions.
9. Review of proper use of digital thermometer, injection-site reaction measurement tool, and memory enhancement card (diary) by study staff during 30-minute post-immunization wait period. Study staff will also discuss signs and symptoms of potential AEs, and indications for use of antipyretics (acetaminophen or ibuprofen) if fever, headache, or malaise occurs.

Study Day 59 ±1 Day (3 Days after Second Vaccination)

1. Perform basic history and focused physical examination (including injection site), emphasizing examination of any acute complaints.
2. Review memory enhancement card.
3. Record vital signs.
4. Obtain blood for CBC with differential and platelet count, ALT, and creatinine.

Study Day 63 ±1 Day (7 Days after Second Vaccination)

1. Perform basic history and focused physical examination (including injection site), emphasizing examination of any acute complaints.
2. Record vital signs.
3. Review memory enhancement card.
4. Obtain urine for dipstick testing.
5. Obtain blood for CBC with differential and platelet count, and B and T cell studies.

Study Day 70±2 Days (14 Days after Second Vaccination)

1. Perform basic history and focused physical examination (including injection site), emphasizing examination of any acute complaints.
2. Record vital signs.
3. Obtain urine for dipstick testing. Obtain urine or serum for β-hCG testing.
4. Obtain blood for CBC with differential and platelet count, ALT, creatinine, anti-Pfs25 antibody ELISA, B and T cell studies, and SMFA.
5. Review pregnancy prevention counseling.

Study Day 84 ±3 Days (28 Days after Second Vaccination)
1. Perform basic history and assessment of any physical complaint.
2. Record vital signs.
3. Obtain urine for dipstick testing.
4. Review pregnancy prevention counseling.

Groups 1(a and b)

Study Day 120 ±7 Days
1. Perform directed history and assessment of any physical complaint.
2. Record vital signs.
3. Obtain urine for dipstick testing.

Study Day 180 ±14 Days
1. Perform directed history and assessment of any physical complaint.
2. Record vital signs.
3. Obtain urine for dipstick testing.
4. Obtain approximately 5 mL of blood for anti-Pfs25 ELISA.

Study Day 270 ±14 Days
1. Perform directed history and assessment of any physical complaint.
2. Record vital signs.
3. Obtain approximately 5 mL of blood for anti-Pfs25 ELISA.

Study Day 420 ±14 Days
1. Perform directed history and assessment of any physical complaint.
2. Record vital signs.
3. Obtain blood for anti-Pfs25 ELISA, and T and B cell studies.

Group 2

Study Day 120 ±28 Days (Day of Third Vaccination)
1. Perform basic history and focused physical examination, emphasizing examination of any acute complaints.
2. Review criteria for vaccination.
3. Obtain blood for CBC with differential and platelet count, ALT, creatinine, anti-Pfs25 antibody ELISA, and B and T cell studies.
4. For females, obtain a urine or serum sample for β-hCG testing. Ensure that the test is negative before vaccinating; a positive test will exclude the subject from the trial. Review pregnancy prevention counseling.
5. Obtain a urine sample for dipstick testing and ensure that protein and blood are no more than trace positive before vaccinating. Should a female subject be menstruating on the day of vaccination, she will not be excluded from vaccination if she has more than trace blood in her urine.

6. Record vital signs (blood pressure, temperature, heart rate, and respiratory rate).
7. Administer the vaccine.
8. Observe for at least 30 minutes after vaccination to evaluate for immediate adverse reactions.
9. Review of proper use of digital thermometer, injection-site reaction measurement tool, and memory enhancement card (diary) by study staff during 30-minute post-immunization wait period. Study staff will also discuss signs and symptoms of potential AEs, and indications for use of antipyretics (acetaminophen or ibuprofen) if fever, headache, or malaise occurs.

Study Day 123 ±1 Day (3 Days after Third Vaccination)
1. Perform basic history and focused physical examination (including injection site), emphasizing examination of any acute complaints.
2. Review memory enhancement card.
3. Record vital signs.
4. Obtain blood for CBC with differential and platelet count, ALT, and creatinine.

Study Day 127 ±1 Day (7 Days after Third Vaccination)
1. Perform basic history and focused physical examination (including injection site), emphasizing examination of any acute complaints.
2. Record vital signs.
3. Review memory enhancement card.
4. Obtain urine for dipstick testing.
5. Obtain blood for CBC with differential and platelet count, and B and T cell studies.

Study Day 134 ±2 Days (14 Days after Third Vaccination)
1. Perform basic history and focused physical examination (including injection site), emphasizing examination of any acute complaints.
2. Record vital signs.
3. Obtain urine for dipstick testing. Obtain urine or serum for β-hCG testing.
4. Obtain blood for CBC with differential and platelet count, ALT, creatinine, anti-Pfs25 ELISA, B and T cells, and SMFA.
5. Review pregnancy prevention counseling.

Study Day 148 ±3 Days (28 Days after Third Vaccination)
1. Perform basic history and assessment of any physical complaint.
2. Record vital signs.
3. Obtain urine for dipstick testing.
4. Review pregnancy prevention counseling.
5. Obtain blood for anti-Pfs25 ELISA

Study Day 175 ±7 Days
1. Perform directed history and assessment of any physical complaint.
2. Record vital signs.
3. Obtain urine for dipstick testing.
4. Obtain blood for anti-Pfs25 ELISA.

Study Day 270 ±14 Days

1. Obtain informed consent for Version 4, if subject is willing.
2. Perform directed history and assessment of any physical complaint.
3. Record vital signs.
4. Obtain blood for CBC with differential and platelet count.
5. Obtain urine for dipstick testing.
6. For females, obtain urine for β-hCG testing.
7. Obtain blood for anti-Pfs25 ELISA.

Group 2 subjects who do not receive optional booster dose

Study Day 480 ±14 Days

1. Perform directed history and assessment of any physical complaint.
2. Record vital signs.
3. Obtain blood for anti-Pfs25 ELISA, and T and B cell studies.

Group 2 and select high-responders in Group 1

Study Day 300 ±14 Days (Day of Third or Fourth Vaccination-approximately 6 months since third vaccination for Group 2, up to 9 months after second vaccination for Group 1)

1. Perform basic history and focused physical examination, emphasizing examination of any acute complaints.
2. Review criteria for vaccination.
3. Obtain blood for CBC with differential and platelet count, ALT, creatinine, anti-Pfs25 antibody ELISA, and B and T cell studies.
4. For females, obtain a urine or serum sample for β-hCG testing. Ensure that the test is negative before vaccinating; a positive test will exclude the subject from vaccination. Review pregnancy prevention counseling.
5. Obtain a urine sample for dipstick testing and ensure that protein and blood are no more than trace positive before vaccinating. Should a female subject be menstruating on the day of vaccination, she will not be excluded from vaccination if she has more than trace blood in her urine.
6. Record vital signs (blood pressure, temperature, heart rate, and respiratory rate).
7. Administer the vaccine.
8. Observe for at least 30 minutes after vaccination to evaluate for immediate adverse reactions.
9. Review of proper use of digital thermometer, injection-site reaction measurement tool, and memory enhancement card (diary) by study staff during 30-minute post-immunization wait period. Study staff will also discuss signs and symptoms of potential AEs, and indications for use of antipyretics (acetaminophen or ibuprofen) if fever, headache, or malaise occurs.

Study Day 303 ±1 Day (3 Days after Booster Vaccination)

1. Perform basic history and focused physical examination (including injection site), emphasizing examination of any acute complaints.
2. Review memory enhancement card.
3. Record vital signs.

4. Obtain blood for CBC with differential and platelet count, ALT, and creatinine.

Study Day 307 ±1 Day (7 Days after Booster Vaccination)
1. Perform basic history and focused physical examination (including injection site), emphasizing examination of any acute complaints.
2. Record vital signs.
3. Review memory enhancement card.
4. Obtain urine for dipstick testing.

Study Day 314 ±2 Days (14 Days after Booster Vaccination)
1. Perform basic history and assessment of any physical complaint.
2. Record vital signs.
3. Obtain urine for dipstick testing.
4. For females, obtain urine or serum for β-hCG testing.
5. Obtain blood for CBC with differential and platelet count, ALT, creatinine, anti-Pfs25 ELISA, B and T cells, and SMFA.
6. Review pregnancy prevention counseling.

Study Day 328 ±3 Days (28 Days after Booster Vaccination)
1. Perform basic history and assessment of any physical complaint.
2. Record vital signs.
3. Obtain urine for dipstick testing.
4. Review pregnancy prevention counseling.
5. Obtain blood for anti-Pfs25 ELISA

Study Day 356 ±7 Days (56 days after Booster Vaccination)
1. Perform directed history and assessment of any physical complaint.
2. Record vital signs.
3. Obtain urine for dipstick testing.
4. Review pregnancy prevention counseling.
5. Obtain blood for anti-Pfs25 ELISA and SMFA.

Study Day 450 ±14 Days (150 days after Booster Vaccination)
1. Perform directed history and assessment of any physical complaint.
2. Record vital signs.
3. Obtain urine for dipstick testing.
4. Obtain blood for anti-Pfs25 ELISA.

Study Day 540 ±14 Days
1. Perform directed history and assessment of any physical complaint.
2. Record vital signs.
3. Obtain urine for dipstick testing.
4. Obtain blood for anti-Pfs25 ELISA.

<u>Study Day 660 ±14 Days</u>

1. Perform directed history and assessment of any physical complaint.
2. Record vital signs.
3. Obtain blood for anti-Pfs25 ELISA, and T and B cell studies.

6.5 Large Volume Blood Draw

Selected subjects with a high antibody response may be invited to undergo an optional large volume blood draw approximately 4 weeks after their third vaccination and/or last vaccination. This will be done under a separate IRB approved protocol and consent (CIR 236-Phlebotomy for collection of Serum, Plasma, and/or Leukocytes for In Vitro Studies). Up to 400 mL of blood will be drawn. Continued participation in the study will NOT be dependent on agreement to undergo this blood draw. Subjects with a hemoglobin <12.5 gm/dL on day 14 after their last vaccination will not be eligible for this blood draw. Subjects will be instructed to eat a large meal and drink several glasses of fluids (caffeine – free) prior to their phlebotomy.

Samples obtained from this blood draw will be used to create a high titer reference standard reagent. Fifty percent of serum per subject derived from this blood draw will be provided, as de-identified samples, to LMVR, a designated laboratory of the PATH Malaria Vaccine Initiative (MVI) for generation of reference standard reagents. This reference serum will be used for research purposes only and will not have any commercial use or value. PBMCs may also be obtained.

6.6 Symptom Memory Enhancement Card

Subjects will be asked to keep daily symptom memory enhancement cards for recording oral temperature once during the day, as well as pain, redness, swelling at the injection site and any systemic signs or symptoms for 6 days following each immunization. The size of any injection-site reaction will be measured using a standardized clear plastic measurement tool and recorded in the memory enhancement card. Clinical site staff will review these cards and record on the source document the daily temperature, injection site erythema, swelling, induration, pain and any other sign or symptom noted by the subject. Systemic symptoms such as fever, headache, nausea, malaise, myalgia, arthralgia and urticaria (see Table 3) will be solicited from the subjects. The memory enhancement cards will not be collected.

6.7 Photographs of Rash or Injection Site Reactions

If a subject develops a rash or injection site reaction, photographs may be taken by the investigators. These photographs will not include the subject's face or any identifying scars, marks, or tattoos.

6.8 Contraindications to Vaccination

The following criteria should be checked prior to each immunization and are contraindications to further immunization. The subject will be encouraged to remain in the safety evaluation for doses already received.

- Hypersensitivity reaction following administration of the study vaccine.

- Positive urine or serum β-hCG test.

- Urine dipstick more than trace positive for blood or protein, confirmed by urinalysis. Should a female subject's urine dipstick show more than trace blood, she will not be excluded from vaccination if she is currently menstruating.

6.9 Indications for Deferral of Vaccination

If any one of the following AEs occurs at the time of the scheduled vaccination, the subject may be either vaccinated at a later date within the allowable time interval specified in the protocol or withdrawn at the discretion of the Investigator:

- Oral temperature >37.5˚C at the time of vaccination will warrant deferral of immunization until fever resolves (within protocol-defined vaccination window).

- Any other condition that in the opinion of the Investigator poses a threat to the individual if immunized or that may complicate interpretation of the safety of vaccine following immunization.

Such individual(s) will be followed in the clinic until the symptoms resolve or the window for immunization expires. No further vaccination will be performed if the subject does not recover (oral temperature ≤37.5˚C and/or lack of symptoms) within the vaccination window. The subject will be monitored for safety and immunogenicity for 12 months after their last vaccination. If the subject does not receive the second or third vaccination, some scheduled safety blood draws may not be performed at the discretion of the PI. Blood draws scheduled to measure immune response will be obtained if possible. If the subject meets any of the above criteria for deferral on the day of first immunization, the Investigator may elect to exclude the subject from further participation in the study. Eligible alternate subjects will be vaccinated instead.

6.10 Subject Withdrawal Criteria

A subject will not be considered to have completed the trial if any of the following reasons apply:

1. *Research terminated by Sponsor or Investigator* – applies to the situation where the entire study is terminated by the Sponsor or Investigator, or other regulatory authority for any reason.
2. *Withdrawal of consent* – applies to a subject who withdraws consent to participate in the study for any reason.
3. *Noncompliant with protocol*- applies to a subject who does not comply with protocol-specific visits or evaluations, on a consistent basis, such that adequate follow-up is not possible and the subject's safety would be compromised by continuing in the trial. This also applies to a subject who is lost to follow-up and is not reachable by telephone or

other means of communication; and is not able to be located.

4. *Developed an AE* - applies to a subject who is withdrawn from study due to an AE, serious or otherwise. Any grade 3 or greater AE that is assessed as possibly, probably, or definitely related to vaccination (other than local reactions lasting <72 hours, or systemic reactions lasting <24 hours) will result in withdrawal of the subject from further vaccinations. Subjects may also be withdrawn for any AE that would cause continued participation in the study to not be in the best interest of the subject, as per the investigator's judgment. Any subject who is withdrawn from the study because of an AE related to study agent will be followed for safety until at least resolution of that AE and will be encouraged to remain in the safety evaluation for the duration of the study.

5. *Other* – is used when previous categories do not apply and a written explanation is required.

If a subject withdraws or is withdrawn prior to completion of the study, the reason for this decision will be recorded in the source documents and CRIMSON Data System. Any subject who has received at least 1 dose of vaccine will be encouraged to remain in the safety evaluation for the duration of the study. The subject's data will be included in the safety and immunogenicity analysis. If a subject fails to complete all planned vaccinations because of an AE or serious adverse event (SAE), the subject will be followed until resolution or stabilization of the event. If a subject withdraws, the investigator will make a reasonable effort to determine the reason.

6.11 Replacement of Subjects

Subjects who have received at least 1 vaccination and who withdraw or are terminated from the study prior to completion will not be replaced.

6.12 Treatments that Could Potentially Interfere with Vaccine-induced Immunity

Treatment with any of the following medications during the study may exclude a subject from receiving further doses of the study vaccine. However, the subject will be encouraged to remain in the study for the duration of the study for safety evaluations.

- Licensed vaccine in the 2-week period (4 weeks for live vaccines) prior to and following each vaccination

- Receipt of immunoglobulins and/or any blood products up to 6 months prior to the first vaccination through 30 days after administration of the last dose of vaccine

- Chronic oral or intravenous administration (≥14 days) of immunosuppressive doses of steroids, i.e., prednisone >10 mg per day, immunosuppressants or other immune-modifying drugs from each day of vaccination to 2 weeks following each vaccination

- Any investigational drug or investigational vaccine other than the study vaccine during the study period

- Required surgical removal of the spleen or the development of a hematologic or other disease that would interfere with normal immunity.

6.13 Clinical Laboratory Testing

Using standard techniques, the clinical laboratory will perform the following tests:

1. Complete blood count (CBC) plus white blood cell differential and platelet count*
2. Serum creatinine
3. Alanine aminotransferase (ALT)
4. HIV assay (Food and Drug Administration [FDA]-approved screening antibody assay with Western Blot
Confirmation)
5. HBsAg ELISA
6. HCV assay (FDA-approved screening antibody assay and immunoblot confirmation or viral PCR confirmation)
7. Urinalysis (in the event of an abnormal urine dipstick test at the clinical trial site)**

* The following CBC parameters will be assessed for safety throughout the trial:
WBC, ANC (absolute neutrophil count), Hemoglobin, and Platelet Count

** Urine and/or serum β-hCG testing will be performed at the clinical trial site using FDA approved products

6.14 Immunologic Laboratory Testing

6.14.1 Antibody Assay (ELISA)

Anti- Pfs25 ELISAs will be performed on sera or plasma obtained from immunized subjects at the Laboratory of Malaria Immunology and Vaccinology (LMIV) in Rockville, MD. Briefly, microwell plates are coated with antigen solution. Plates are washed with TRIS-buffered saline (TBS) containing Tween-20 (T-TBS) and blocked with TBS containing skim milk powder. After washing with T-TBS, diluted serum samples are added in triplicate and incubated at room temperature for 2 hours. After incubation, unbound antibodies are removed by washing the plates with T-TBS, and alkaline phosphatase-conjugated goat anti-human IgG solution is added to each well and incubated for 2 hours at room temperature. Plates are then washed with T-TBS, followed by adding phosphatase substrate solution to each well; the plates are then covered and incubated for 20 minutes at room temperature for color development. The plates are read immediately at 405 nm with a microplate reader. The optical density values are used to determine antibody levels by comparing to a standard curve generated from a known positive-control plasma included on each ELISA plate.

6.14.2 Standard Membrane Feeding Assay (SMFA)

Membrane feeding assays demonstrate biologic activity of transmission blocking antibody, and

are critical to selection of vaccine candidates. In a SMFA, test serum or plasma obtained from immunized subjects is mixed with parasites from a laboratory culture and the mixture is placed in a feeding cup covered with an artificial membrane. Pre-starved mosquitoes from a laboratory colony are allowed to feed through the membrane. One week after the feed, mosquitoes are dissected and midguts are stained with mercurochrome for the oocyst form of the parasite. The reduction of oocyst-laden mosquitoes or the reduction of oocyst numbers in each mosquito demonstrate biologic function of the antibody, and may be predictive of efficacy in the field. SMFA results have been shown to correlate with ELISA antibody titers against Pfs25 in several species [Cheru et al 2010].

The assay will be conducted at LMIV and the Laboratory of Malaria Vector Research (LMVR), Rockville, MD.

Additional exploratory direct membrane feeding assays will be done by the Entomology Department, Armed Forces Research Institute of Medical Sciences, Bangkok, Thailand by the Malaria Research and Training Center, Universite de Bamako, Bamako, Mali, and by the Institut de Recherche en Sciences de la Santé (IRSS), Bobo-Dioulasso, Burkina Faso (via the Institut de Recherche pour le Développement (IRD) - Montpellier, France). The assay may use gametocytes from volunteers recruited under separate local IRB-approved protocols and consents.

6.14.3 B-Cell and T-Cell Assays

Specimens collected for B cells and T cells studies will undergo initial processing and cell separation at the CIR, and will be shipped to LMIV in liquid nitrogen shippers as per SOP.

B cell studies will be done at LMIV. The analysis of the generation and maintenance of antigen-specific memory B cells will be carried out to determine if these cells can be elicited and maintained by vaccination. Peripheral blood lymphocytes will be obtained and assayed for the presence of antigen-specific memory B cells and for the total number of memory B cells using flow cytometry and ELISPOT assays.

T cell studies will be done at LMIV. Antigen-specific T cell responses to vaccination will be determined by ELISPOT and/or Intracellular Cytokine Staining flow cytometry.

7 Use, Storage, and Tracking of Specimens and Data

Samples and data collected under this protocol will be used to study malaria and related diseases, and possible adverse reactions to vaccination. Access to research samples will be limited using either a locked room or a locked freezer. Samples and data will be stored using codes assigned by the investigators or their designees. Data will be kept in password-protected computers. Only investigators or their designees will have access to the samples and data.

Samples will be stored at the LMIV in Rockville, MD or at LMIV's designated repository, Thermo Scientific, Rockville, MD with the exception of retention specimens which may be kept at the CIR for quality control. Samples may also be stored at LMVR, NIAID, a laboratory

designated by the PATH Malaria Vaccine Initiative. Samples will be tracked using a sample tracking software program, e.g., Freezerworks. Any loss or unanticipated destruction of samples (for example, due to freezer malfunction) or data (for example, misplacing a printout of data with identifiers) will be reported to the IRBs. Such a loss will be reported to the NIAID IRB as a protocol violation under the following classification: the violation compromises the scientific integrity of the data collected for the study.

8 Retention of Specimens for Future Use

Specimens collected as part of this trial will be stored for future research. These samples may be used to learn more about malaria infection and other diseases. These samples will not be sold or used to make commercial products. The subject may withdraw permission for future use of specimens at any time. If a subject withdraws his or her permission for future use of specimens, those specimens will be destroyed. All samples stored will be labeled with the subject's study identification (ID) number, which cannot identify the study subject but is linkable to other research databases (e.g., questionnaires, clinical assessments, logbooks) generated by the main study. The database will contain only the study subject's ID number. A master log linking the study subject ID number to the name of the subject will be maintained in a password protected database system with access limited to authorized research team members. In the event of samples being requested in the future, only the site Investigators or site study coordinator will have access to the log linking the study subject to the samples.

At the completion of the protocol (termination), samples and data will either be destroyed, or transferred to another existing protocol, "Research Use of Human Specimens", NIAID Protocol #08-I-N064. In the future, other investigators may wish to study these samples and/or data. If so, the National Institutes of Health (NIH) and/or the PATH Malaria Vaccine Initiative may send samples to the other investigators. In that case, IRB approval must be sought prior to any sharing of samples. Any clinical information shared about the sample with or without patient identifiers would similarly require prior IRB approval. The research use of stored, unlinked, or unidentified samples (for example, as a standard for immunological analyses) may be exempt from the need for prospective IRB review and approval. Exemption requests will be submitted in writing to the NIH Office of Human Subjects Research (OHSR), which is authorized to determine whether a research activity is exempt.

9 Safety and Adverse Event Reporting

9.1 Definitions

Adverse Events

An adverse event (AE) includes any untoward or unfavorable medical occurrence in a human subject, including any abnormal sign (e.g. abnormal physical exam or laboratory finding), symptom, or disease, temporally associated with the subject's participation in the research, whether or not considered related to the research. This includes an exacerbation of pre-existing conditions and intercurrent illnesses. All AEs will be graded for severity and assessed for

relationship to the investigational vaccine as described below.

Adverse Reactions
An adverse reaction is an AE that is caused by the vaccine.

Suspected Adverse Reaction (SAR)
An adverse event for which there is a reasonable possibility that the drug caused the adverse event. ‘Reasonable possibility’ means that there is evidence to suggest a causal relationship between the drug and the adverse event. A suspected adverse reaction implies a lesser degree of certainty about causality than adverse reaction which implies a high degree of certainty.

Serious Adverse Events
A Serious Adverse Event (SAE) is defined as an AE that results in any of the following outcomes:

- death
- life threatening (i.e., an immediate threat to life)
- inpatient hospitalization or prolongation of an existing hospitalization
- a persistent or significant incapacity or substantial disruption of the ability to conduct normal life functions
- congenital anomaly/birth defect
- other medically important event*

* Medical and scientific judgment will be exercised in deciding whether expedited reporting is appropriate in other situations, such as important medical events that may not be immediately life threatening or result in death or result in hospitalization but may jeopardize the patient or may require intervention to prevent one of the other outcomes listed above. These will also usually be considered serious.

Unexpected Adverse Events
An AE is considered unexpected if it is not listed in the Investigator Brochure or Package Insert (for marketed products) or is not listed at the specificity or severity that has been observed. ”Expected” does not mean that the event is expected with pharmacologically similar drugs, the underlying disease(s) or concomitant medications.

Suspected and Unexpected Serious Adverse Reaction (SUSAR)
A SUSAR is a Suspected Adverse Reaction that is both Serious and Unexpected. Under 21 CFR 312.32, SUSARs will be reported by the IND Sponsor to the FDA and to all participating investigators in an IND Safety Report.

New Onset of Chronic Illness (NOCI)
The new onset of chronic illness is defined as a diagnosis of a new medical condition that is chronic in nature, including those potentially controllable by medication (e.g., diabetes, asthma). Any NOCI will be recorded in the same manner as unsolicited AEs.

Unanticipated Problems
An Unanticipated Problem (UP) is any incident, experience, or outcome that is

1. unexpected in terms of nature, severity, or frequency in relation to
   a. the research risks that are described in the IRB-approved research protocol and informed consent document; Investigator's Brochure or other study documents; and
   b. the characteristics of the subject population being studied; and
2. related or possibly related to participation in the research; and
3. places subjects or others at a greater risk of harm (including physical, psychological, economic, or social harm) than was previously known or recognized. (An AE with a serious outcome will be considered increased risk.)

Unanticipated problem that is not an Adverse Event (UPnonAE)
An UP that does not fit the definition of an AE, but which may, in the opinion of the investigator, involve risk to the subject, affect others in the research study, or significantly impact the integrity of research data; for example, occurrences of breaches of confidentiality, accidental destruction of study records, or unaccounted-for study drug.

Protocol Violation
Any change, divergence, or departure from the study procedures in an IRB-approved research protocol that has a major impact on the subject's rights, safety, or well-being and/or the completeness, accuracy or reliability of the study data.

Protocol Deviation
Any change, divergence, or departure from the IRB approved study procedures in a research protocol that **does not** have a major impact on the subject's rights, safety or well-being, or the completeness, accuracy and reliability of the study data.

9.2 Preexisting Conditions, Conditions, Worsening of Pre-existing Condition

Stable chronic conditions which are present prior to enrollment and do not worsen are not considered AEs and will be accounted for in the subject's medical history. Exacerbation or worsening of pre-existing conditions are defined as AEs and are evaluated using the same criteria described in **Section 9.3.2** in this protocol.

9.3 Assessment of Adverse Events

Assessment of safety will include clinical observation and monitoring of hematological, chemical, and immunologic parameters. Subjects will be closely monitored for 30 minutes following each immunization. Additionally, subjects will return to the clinic on Days 3, 7, 14, and 28 following each vaccination for clinical assessments, and periodically thereafter until completion. All AEs will be recorded through Day 28 after each vaccination. Injection site reactions will be assessed until day 14 or until resolved. After that period only SAEs, UPs, and NOCIs (Section 9.1) will be recorded.

All AEs will be graded for severity and assessed for relationship to the study product. Reactions

will be graded as described in this protocol. A study clinician will be available 24 hours a day during the study period. Should a subject call a study clinician to report an AE, it will be fully documented in the subject's study chart, and discussed with the PI.

All local and systemic reactions will be captured on the appropriate source documents and CRIMSON Data System. Those assessed as serious will be further reported on the Sponsor's SAE/UP report form. AEs judged to be possibly, probably, or definitely related to the study product will be followed to adequate resolution. All concomitant medications will be collected through day 28 after each vaccination; new chronic medications will be collected for the remainder of the study.

9.3.1 Adverse Event Definitions

Headache: a pain located in the head, over the eyes, at the temples, or at the base of the skull lasting more than 30 minutes
Nausea: discomfort in the stomach with an urge to vomit
Malaise: generalized feeling of being unwell
Myalgia: pain in the muscles, in one or more muscle groups
Arthralgia: pain in a joint, in one or more joints
Urticaria: Hives; a raised, red, itchy skin rash containing wheals

Table 3 Solicited Adverse Events

Systemic adverse events	Laboratory adverse events	Local reactogenicity
Fever	Hemoglobin	Injection pain
Headache	WBC	Injection erythema
Nausea	ANC	Injection swelling
Malaise	ALT	Injection induration
Myalgia	Platelet count	Injection pruritus
Arthralgia	Creatinine	
Urticaria		

9.3.2 Determination of Severity

Severity of AEs will be assessed by the investigator as described in Appendix C. AEs not included in the Appendixes will be graded for severity using the followings definitions:

Grade 1 (Mild): No interference with activity, may use 1 dose of an over the counter medication

Grade 2 (Moderate): Repeated use of non-narcotic pain reliever > 24 hours or some interference with activity

Grade 3 (Severe): Activities of daily living limited to <50% of baseline, medical evaluation/therapy required

Grade 4 (Life-threatening):	Extreme limitation in activity, significant assistance required; immediate medical intervention or therapy required to prevent death
Grade 5:	Death

9.3.3 Association with Receipt of the Study Vaccine

All AEs will be assessed for relationship to the study vaccine using the following definition:

Definitely:	Clear-cut temporal association, and no other possible cause.
Probably:	Clear-cut temporal association and a potential alternative cause is not apparent.
Possibly:	Less clear temporal association; other causes also possible.
Unlikely:	Temporal association between the AE and the vaccine or the nature of the event is such that the vaccine is not likely to have had any reasonable association with the observed illness/event (cause and effect relationship improbable but not impossible).
Not Related:	The AE is completely independent of vaccine administration; and/or evidence exists that the event is definitely related to another cause.

The degree of certainty with which an AE can be attributed to administration of the study vaccine will be determined by how well the event can be understood in terms of one or more of the following:

1. The event being temporally related with vaccination or reproduced on re-vaccination
2. A reaction of similar nature having previously been observed with this type of vaccine and/or formulation
3. The event having been reported in the literature for similar types of vaccines
4. Whether or not there is another identifiable cause

All local (injection-site) reactions will be considered causally related to vaccination.

Reports will further classify AEs as follows: Related (all AEs that are assessed as definitely, probably, or possibly related) and Unrelated (all AEs assessed as unlikely or definitely not related), as per Table 4 below. When reporting to regulatory authorities and IRBs is needed, AE relationship will be determined per Table 4.

Table 4 Assessment of Relationship

Related			Not related	
Definitely	Probably	Possibly	Unlikely	Not related

9.4 Reporting

9.4.1 Reporting to IND Sponsor

SERIOUS ADVERSE EVENTS

SAEs will also be reported on the SAE/Unanticipated Problems Report Form and sent to the Sponsor Clinical Safety Office (CSO) by fax or e-mail attachment. All SAEs will be reported within 1 business day after the clinical site becomes aware of the event.

UNANTICIPATED PROBLEMS

Unanticipated Problems that are AEs will also be reported on the SAE/Unanticipated Problems Report Form and sent to the Sponsor Clinical Safety Office (CSO) by fax or e-mail attachment no later than 14 calendar days of PI awareness of the event. Unanticipated Problems that are not AEs will not be reported to the Sponsor CSO.

Unanticipated AEs will also be reported to the regulatory authorities and IRBs per relevant FDA and IRB guidelines. When an unanticipated AE occurs, the relationship to study product will be assessed per Table 4 and reported accordingly.

SPONSOR CLINICAL SAFETY OFFICE CONTACT INFORMATION:

RCHSPB Clinical Safety Office
5705 Industry Lane
Frederick, MD 21704

Phone 301-846-5301
Fax 301-846-6224
E-mail: rchspsafety@mail.nih.gov

Pregnancy

Pregnancy itself is not an AE. However, pertinent obstetrical information for all pregnancies will be reported to the CSO via fax or email within 3 business days from Investigator awareness of the pregnancy.

Pregnancy outcome data (e.g., delivery outcome, spontaneous, or elective termination of the pregnancy) will be reported to the CSO within 3 business days of the site's awareness of the outcome on a protocol-specified form.

In the event of pregnancy the following steps will be taken:

- Withdraw from the study but continue in follow up for safety
- Report to Western and NIAID IRBs as an unanticipated problem
- Advise research participant to notify the obstetrician of study vaccine exposure

SPONSOR'S REPORTING RESPONSIBILITIES

Serious, unexpected, suspected adverse reactions (SUSARs) as defined in 21 CFR 312.32 will be reported to FDA and all participating Investigators as IND Safety Reports. The sponsor will also submit a brief report of the progress of the investigation to the FDA on an annual basis as defined in 21 CFR 312.33.

9.4.2 Reporting to the IRBs

Reporting requirements to the NIAID Institutional Review Board (IRB) for this protocol are as follows:

Expedited reporting:
Unanticipated problems that are either AEs or non-AEs (as defined above) and protocol violations will be reported within 7 calendar days of investigator awareness. SAEs that are possibly, probably, or definitely related to the research will be reported within 7 calendar days of investigator awareness, regardless of expectedness.

Annual reporting:
The following items will be reported to the NIAID IRB in summary at the time of Continuing Review:

- All unanticipated problems
- All protocol deviations which, in the opinion of the investigator, will be reported
- All SAEs (related and unrelated, and expected and unexpected).

All SAEs, unanticipated problems, AEs, and protocol violations and deviations will be reported to Western Institutional Review Board (WIRB) as per WIRB guidelines, below:

- **WIRB Guidelines:**
  WIRB Phone: 800-562-4789, Fax: 360-252-2498
  http://www.wirb.com/ for updated reporting guidelines

Reports for continuing review to WIRB will be submitted by the investigators per WIRB guidelines. WIRB is the site IRB and as such is the IRB of record for this study.

9.4.3 Reporting to Malaria Vaccine Initiative

All SAEs will be reported to Malaria Vaccine Initiative (MVI) within 24 hours of investigator awareness. Unanticipated problems and AEs will be reported to MVI at the same time they are reported to WIRB.

MVI contact:

Didier Leboulleux
PATH Malaria Vaccine Initiative
Batiment avant-centre, 13 Chemin du Levant, 01210 Ferney-Voltaire, France
Direct phone: +33 (0) 4.50.28.62.80
Mobile: +33 (0) 6 33 04 52 40
fax: +33 (0) 4.50.28.04.07

Email: dleboulleux@path.org

9.4.4 Reporting to the Safety Monitoring Committee

SAEs reported to the Regulatory Compliance and Human Subjects Protection Branch (RCHSPB) CSO will be reported to the Safety Monitoring Committee (SMC) Executive Secretary by email or fax according to the RCHSPB SMC Policy. Should halting rules be met the SMC will be notified and a safety review will be scheduled. All AEs will be summarized and forwarded to the CSO Executive Secretary for distribution to the SMC prior to the review. The AE's relationship to study product for this purpose will be categorized according to Table 4.

9.5 Halting Rules

The PI will closely monitor study data as they become available and will make determinations regarding the presence and grading of AEs. The AEs will be evaluated with regard to the known complications associated with administration of vaccine components. If a dose of vaccine is considered unacceptably reactogenic (as described in the following criteria), the study will be halted. No new enrollments and no further vaccinations will be administered by the Investigators until reviewed by the SMC and study IND Sponsor. A report of SMC recommendations will be submitted to the IRBs. The following criteria will be used to define unacceptable reactogenicity of the malaria vaccine (AEs that are possibly, probably, or definitely related to the vaccine will be considered "Related" as per Table 4 and will be summarized as such):

1. One or more subjects experience an SAE as defined in Section 9.1 of this protocol that is determined to be possibly, probably, or definitely related to the vaccine, **or**
2. One or more subjects experience a hypersensitivity reaction that is probably or definitely related to the vaccine, **or**
3. Any severe clinical illness occurs that is not explained by a diagnosis that is unrelated to vaccination, **or**
4. Two or more subjects in any dose cohort experience any Grade 2 or higher laboratory abnormality (see Appendix C), or Grade 3 systemic AE that is determined to be possibly, probably, or definitely related to the vaccine as defined in this protocol, **or**
5. One or more subjects experience erythema nodosum.
6. Two or more volunteers in a single dose cohort experience Grade 3 or higher injection site induration or any abscess formation.

The IRBs, the NIAID, the FDA, or other government agencies may discontinue the study at any time. Subsequent review of serious, unexpected, and related AEs by the SMC or IRB, the IND sponsor, the FDA, and other regulatory authorities may also result in suspension of further administration of vaccine at the clinical site. The FDA, other regulatory authorities, and the study sponsor(s) retain the authority to suspend additional enrollment and administration of vaccine for the entire study as applicable.

10 Clinical Monitoring

10.1 Site Monitoring Plan

RCHSPB will provide oversight and monitor the compliance of this trial. Monitors under contract to the NIAID/RCHSPB will visit the clinical site to monitor all aspects of the trial in accordance with appropriate regulations. The objectives of a monitoring visit will be:

1. to verify the prompt reporting of all data points, including SAEs
2. to check the availability of signed informed consent documents and documentation of the ICF process for each monitored subject
3. to compare individual subject's records (e.g. CRFs, electronic data, CRIMSON pulls) to the source documents (supporting data, laboratory specimen records, clinical notes)
4. to ensure the investigators are in compliance with the protocol, and accuracy and completeness of records

The monitors will also inspect the clinical site's regulatory files to ensure that applicable regulatory requirements (FDA, Office for Human Research Protections [OHRP]) and guidelines (ICH) are being obeyed. During the monitoring visits, the PI and/or designated study staff will be available to discuss the study. The site PI will provide direct access and allow the study monitors, LMIV, and regulatory authorities to access all study-related documents.

Prior to the start of the study, the PIs will be informed of the frequency of the monitoring visits and will be given reasonable notification prior to each visit.

Quality control procedures will be implemented beginning with the data entry system, and data quality control checks that will be run on the database will be generated. Any missing data or data anomalies will be communicated to the site for clarification/resolution.

MVI will have the right to audit the site. During the audit the PI and/or designated study staff will be available to discuss the study. The site PI will provide direct access and allow the MVI representative access to all study-related documents.

10.2 Safety Monitoring Plan

10.2.1 Safety Monitoring Committee (SMC)

An independent Safety Monitoring Committee (SMC) will be formed in accordance with RCHSPB guidelines. The SMC will review the study protocol and safety monitoring plan prior to enrollment and will review safety data should halting rules be met, or at the Investigators' discretion. Written summaries of all SMC meetings and recommendations will be submitted to the IRBs as part of annual continuing review. If halting rules are met or clinical events affecting risk occur, meeting minutes and recommendations will be sent to the NIAID IRB as an informational item. Otherwise such reports will be submitted as part of the annual continuing review.

11 Statistical Considerations

11.1 Description of Statistical Methods

This study, like other Phase 1 studies, is exploratory rather than confirmatory; its purpose is to estimate AE rates and patterns of immune responses rather than to test formal statistical hypotheses. Estimates will be presented with their 95% confidence intervals. Descriptive approaches will be used to meet the protocol objectives, as well as formal statistical tests as outlined below. Results will be presented in tabular format, as well as graphically where appropriate.

11.2 Primary Objective

The primary objective of this study is to assess safety and reactogenicity of Pfs25-EPA/Alhydrogel® in malaria-naïve US adults.

a. The frequency of systemic and local AEs will be summarized.
b. A line listing of each clinical and laboratory AE classified as local, solicited, or other will be displayed in tables stratified by vaccine allocation.
c. AEs will be summarized by severity and relationship to vaccine (according to Table 4).
d. The proportion of subjects with at least 1 AE will be compared by vaccine schedule and/or dose group, and tests performed to assess whether these groups differ with respect to these proportions. To see if there is a difference in AEs between the initial vaccination and the subsequent vaccinations, a Wilcoxon signed rank test will be performed, where the response for each subject is the difference between the numbers of AEs in the 2 weeks following each vaccination.
e. SAEs occurring within the study period will be listed by relationship to vaccine.

11.3 Secondary Objective

The secondary objective of this study is to determine the antibody response of the Pfs25 protein vaccines as measured by ELISA and transmission blocking assays as measured by SMFA, and the effect on antibody responses of a third (booster) dose at 4 months. An additional objective is the effect on antibody responses of a longer term booster (third or fourth dose) at 10 months (approximately 6 to 9 months after last dose).

Anti-Pfs25 antibody will be measured by ELISA on vaccination days, 2 weeks after each vaccination, and periodically until study completion. Antibody responses over time will be shown graphically with geometric mean antibodies and the associated confidence intervals based on the t-distribution applied to the log transformed responses. While Group 1 is included primarily for safety, we will still be able to examine a dose effect.

- Dose effect: Here we can compare the antibody responses at 2 weeks after the second

vaccination using all 3 Groups. We use a Jonkheere-Terpstra test (which is a generalization of the Wilcoxon Mann Whitney test for the case when there are three or more ordered groups), to test for the dose effect.

Graphs will display transmission blocking assays as a function of ELISA antibody levels.

11.3.1 Exploratory Objectives

- To determine T- and B-cell responses to vaccination.

Should the study be terminated early, the investigative team will discuss with the SMC the reason for termination and determine which study questions can be addressed in an unbiased manner with the available data. The available data will be analyzed and interpreted in light of early termination.

11.4 Sample Size and Power Calculations

The study is powered to provide sufficient safety data before dose escalation (between Groups 1a, 1b, and Group 2) and before proceeding to a Phase 1 study in a malaria-exposed population. Group 1 is sized for safety, as the higher dose is expected to be necessary for an adequate immune response. In Groups 1a and 1b, 5 subjects will receive 8 and 16 μg Pfs25-EPA/Alhydrogel®, respectively. Vaccination of 5 subjects gives a probability of at least 0.80 for detecting one or more serious or severe AEs that occurs with a probability of 0.275 or more per subject. For the study as a whole, vaccination of 30 subjects gives a probability of at least 0.80 for detecting one or more serious or severe AEs that occurs with a probability of 0.052 or more per subject.

To test for an antibody response by dose between the three dose groups, we use the Jonckheere-Terpstra (J-T) test which is a generalization of the Wilcoxon Mann Whitney (WMW) test from two groups to three or more ordered groups (e.g., low, middle and high dose groups). For the sample size calculations, we consider the simpler situation where the lower two dose groups are combined into one group and tested by WMW test. This simpler situation ignores differences in antibody response between the two lower dose groups; therefore the actual J-T test which does not ignore those differences should be at least as powerful as the simplifying situation. For the power calculation for the simplified situation we first estimate the standard deviation of the (log transformed) antibody response. To do this, we use data on the 5 subjects who received 2 doses of Pfs25/ISA51 vaccine in [Wu et al 2008]. From that data the standard deviation of the log transformed ELISA responses 2 weeks after the second vaccination was 0.33. After accounting for the fact that the standard deviation was estimated from only 5 observations [Fay et al 2007], a Z-test of the difference in means with 4 and 16 observations would have about 80% power to reject at the 2-sided 0.05 level if the geometric mean ELISA values was 4.5 fold higher in the larger dose group (the group with n=16). Because we use the WMW test instead of a Z-test, these results should hold with an increase in the sample size by 5% since the WMW test is about 95.5% as efficient as a Z-test in this situation [Lehmann 1999], i.e., the sample sizes of 10 (low and middle dose combined) and 20 (high dose) will have about 80% power to show a 3 fold difference between the lower two doses and the high dose. Thus, when we use the information

about differences between the low and middle dose groups (i.e., use the J-T test), we should have at least 80% power if there was a difference of 3 fold between the middle and high dose and possibly a larger difference between the low dose and high dose.

11.5 Randomization

Randomization for Group 2 will be done in blocks by the study site pharmacist. Subjects and investigators will be aware of Group assignment. (Note: subjects in Group 2 were initially randomized into Group 2a (0, 2 month schedule) and Group 2b (0, 2, 4 month schedule), but these groups were subsequently collapsed into Group 2 (0, 2, 4 month schedule.) A fourth dose was later added for Group 2 and selected high responders in Group 1 at 10 months.

12 Human Subject Protections and Ethical Obligations

This research will be conducted in compliance with the protocol, Good Clinical Practices (GCP), and all applicable regulatory requirements.

12.1 Institutional Review Board

A copy of the protocol, informed consent forms, and other information to be completed by subjects, such as questionnaires, and any proposed advertising/recruitment materials or letters to the subjects will be submitted to the reviewing IRBs for written approval. The investigator must submit and obtain approval from the IRBs for all subsequent amendments to the protocol, informed consent documents, and other study documentation referenced above. The investigator will be responsible for obtaining IRB approval of the annual Continuing Review throughout the duration of the study. The investigators will notify the reviewing IRBs of protocol violations and SAEs as specified in the relevant sections of the protocol.

12.2 Informed Consent Process

Informed consent is a process that is initiated prior to the individual's agreeing to participate in the study and continuing throughout the individual's study participation. Extensive discussion of risks and possible benefits of this therapy will be provided to the subjects. Consent forms describing in detail the study agent/intervention(s), study procedures, and risks will be given to the subject, and written documentation of informed consent is required prior to starting study agent/intervention. Consent forms will be approved by all participating IRBs, and the subject will be asked to read and review the document. Upon reviewing the document, the investigator will explain the research study to the subject and answer any questions that may arise. The subjects will sign the informed consent document prior to any procedures being done specifically for the study. The subjects will have sufficient opportunity to discuss the study and process the information in the consent process prior to agreeing to participate. The subjects may withdraw consent at any time throughout the course of the trial.

The informed consent process will be documented in the subject's research chart, as required by 21 CFR 312.62. The informed consent form will be signed and personally dated by the subject and the person who conducted the informed consent discussion. The original signed informed

consent form will be retained in the subject's chart and a signed and dated copy will be provided to the subject.

12.3 Justification for Exclusion of Children

This study will not enroll children, since safety has not yet been established in adults.

12.4 Subject Confidentiality

Subjects will not be identified in any publicly released reports of this study. All records will be kept confidential to the extent provided by federal, state, and local law. The study monitors and other authorized representatives of the Sponsor may inspect all documents and records required to be maintained by the Investigator. The investigator will inform the subjects that the above-named representatives will review their study-related records without violating the confidentiality of the subjects. All laboratory specimens, evaluation forms, reports, and other records that leave the site will be identified only by a coded number in order to maintain subject confidentiality. All records will be kept locked and all computer entry and networking programs will be done with coded numbers only. Clinical information will not be released without written permission of the subject, except as necessary for monitoring by IRB, the FDA, the NIAID, the OHRP or the sponsor's designee.

12.5 Risks

Risks to the subjects are associated with venipuncture, immunization, and large volume blood drawing. These risks are outlined below:

12.5.1 Venipuncture

Risks occasionally associated with venipuncture include pain, bruising, bleeding and infection at the site of venipuncture, lightheadedness, and rarely, syncope.

12.5.2 Immunization

Possible local vaccine reactions include pain, swelling, erythema, induration, limitation of limb movement for several days, lymphadenopathy, or pruritus at the injection site. Local subcutaneous (SQ) nodules, believed to be granulomatous reactions to aluminum, have been observed with use of aluminum-based adjuvants. Thus, most aluminum-absorbed vaccines are injected intramuscularly (IM) rather than SQ. Systemic reactions such as fever, chills, headache, fatigue, malaise, myalgia, and joint pain may also possibly occur, with some reactions moderate or severe.

As with any vaccine, immediate hypersensitivity reactions including urticaria, anaphylaxis, or other IgE-mediated responses are possible. There is a theoretical possibility of risks about which we have no present knowledge. Subjects will be informed of any such risks should further data become available.

Subjects may be asked to defer routine immunization (such as influenza) until 14 days following

vaccination. This may increase the risk that the subject will be infected with an influenza virus during this period.

12.6 Benefits

Subjects will not receive any direct benefit from participation in this study. It is hoped that information gained in this study will contribute to the development of a safe and effective malaria vaccine.

12.7 Compensation

Subjects will be paid $80.00 for their screening visit and $80.00 per visit during participation in the study. On vaccination days the subjects will be compensated $125.00 because they are required to stay in the clinic for a substantially longer period of time. Subjects will be paid for the screening visit and for each clinic visit if enrolled. Subjects will only be compensated for screening if they are enrolled in the trial. Subjects who attend as alternates on the day of first vaccination but are not enrolled, will receive payments for the screening visit and the vaccination day visit. Two additional payments totaling up to $650.00 will be paid for completion of all visits – deductions for any missed visits will be taken from these payments. Subjects who complete the fourth or booster dose and its associated follow-up visits will get an additional $300, with deductions for any missed visits. The total payment: up to $1,940 for Groups 1a and b (up to $2,925 if they receive the extra booster dose) and up to $3,290 for Group 2 will be divided over the course of the study with 1 of the additional payments being dispensed midway through the trial and the second dispensed upon completion of the trial (or Day 300 for those going on to a booster dose). The third additional payment will be dispensed at the completion of the trial for those receiving the booster dose.

13 Data Handling and Record Keeping

13.1 Source Documentation

Complete source documentation (laboratory test reports, hospital or medical records, progress notes, observations, subject diaries, etc.) is required for every study subject for the duration of the study. The subject's research record must record his/her participation in the clinical trial, the treatment received (with doses and frequency) or other concomitant medications or interventions administered, as well as any adverse reactions experienced during the trial. Selected data from source documentation and subject symptom diaries for subjects enrolled in the study will be entered into the CRIMSON Data System. The data entry is to be completed on an ongoing basis during the study. Data entered into CRIMSON shall be performed by authorized individuals. Corrections to the data system shall be tracked electronically (password protected) with time, date, individual making the correction, and what was changed. Source documentation should support the data collected in CRIMSON**,** and must be signed and dated by the person recording and/or reviewing the data.

The Investigator is responsible for the accuracy, completeness and timeliness of the data reported

to the Sponsor in the CRIMSON Data System. All data entered into CRIMSON should be reviewed by the Investigator and signed as required with written or electronic signature, as appropriate. Data reported in CRIMSON should be consistent with source documents or the discrepancies should be explained. Source documentation will be made available for review or audit by the Sponsor, MVI or their designees and any applicable Federal authorities.

13.2 Retention of Study Records

The investigator is responsible for retaining all essential documents listed in the ICH Good Clinical Practice Guideline. All essential documentation for all subjects are to be maintained by the investigators in a secure storage facility for a minimum of 3 years, per DHHS (45 CFR 46.115(b)). The FDA requires study records to be retained for up to 2 years after marketing approval or disapproval (21 CFR 312.62), or until at least 2 years have elapsed since the formal discontinuation of clinical development of the investigational agent for a specific indication. These records are also to be maintained in compliance with IRB/EC, state, and federal medical records retention requirements, whichever is longest. All stored records are to be kept confidential to the extent provided by federal, state, and local law. It is the investigator's responsibility to retain copies of source documents until receipt of written notification to the contrary from the RCHSPB of the National Institute of Allergy and Infectious Diseases (NIAID). No study document should be destroyed without prior written agreement between RCHSPB/NIAID and the PI. Should the investigator wish to assign the study records to another party and/or move them to another location, the investigator must provide written notification of such intent to RCHSPB/NIAID with the name of the person who will accept responsibility for the transferred records and/or their new location. NIAID must be notified in writing and written NIAID permission must be received by the site prior to destruction or relocation of research records.

13.3 Protocol Revisions

No revisions to this protocol will be permitted without documented approval from the IRBs that granted the original approval for the study. Any change to the protocol will be submitted to the sponsor and to the participating IRBs as a protocol amendment; and changes not affecting risk to subjects may request an expedited review. In the event of a medical emergency, the Investigator shall perform any medical procedures that are deemed medically appropriate and will notify the IND Sponsor of all such occurrences.

References

1. Birkett AJ. (2010) PATH Malaria Vaccine Initiative (MVI): perspectives on the status of malaria vaccine development. Hum Vaccin. 6(1):139-45.
2. Cheru L, Wu Y, Diouf A, Moretz SE, Muratova OV, Song G, Fay MP, Miller LH, Long CA, Miura K. (2010) The IC(50) of anti-PFS25 antibody in membrane-feeding assay varies among species. Vaccine. 28(27):4423-9.
3. Coler RN, Baldwin SL, Shaverdian N, Bertholet S, Reed SJ, Raman VS, Lu X, DeVos J, Hancock K, Katz JM, Vedvick TS, Duthie MS, Clegg CH, Van Hoeven N, Reed SG. (2010) A synthetic adjuvant to enhance and expand immune responses to influenza vaccines. PLoS One. Oct 27;5(10):e13677.
4. Fay, MP, Halloran, ME, and Follmann, DA. (2007). Accounting for variability in sample size estimation with applications to nonadherence and estimation of variance and effect size. Biometrics 63: 465-474.
5. Eisenbarth SC, Colegio OR, O'Connor W et al. (2008) Crucial role for the NALP3 inflammasome in the immunostimulatory properties of aluminium adjuvants. *Nature* **453**, 1122-1126.
6. Fox CB, Friede M, Reed SG, Ireton GC. (2010) Synthetic and natural TLR4 agonists as safe and effective vaccine adjuvants. Subcell Biochem.53:303-21.
7. Greenwood B, Targett G. (2009) Do we still need a malaria vaccine? Parasite Immunol. 31(9):582-6.
8. Kaslow DC (2002) Transmission-blocking vaccines. Chem Immunol 80: 287–307.
9. Kubler-Kielb J, Majadly F, Wu Y, et al. (2007) Long-lasting and transmission-blocking activity of antibodies to Plasmodium falciparum elicited in mice by protein conjugates of Pfs25. Proc. Natl. Acad. Sci. USA. 104(1):293-8.
10. Lehmann, EL (1999). Elements of Large Sample Theory. Springer: New York, p. 177.
11. Lin FY, Ho VA, Khiem HB, Trach DD, Bay PV, Thanh TC, Kossaczka Z, Bryla DA, Shiloach J, Robbins JB, Schneerson R, Szu SC. (2001) The efficacy of a Salmonella typhi Vi conjugate vaccine in two-to-five-year-old children. N Engl J Med. Apr 26;344(17):1263-9.
12. Malkin EM, Durbin AP, Diemert DJ, et al. (2005) Phase 1 vaccine trial of Pvs25H: a transmission blocking vaccine for Plasmodium vivax malaria.Vaccine. 2005 May 2;23(24):3131-8.
13. Qian F, Wu Y, Muratova O, et al. (2007) Conjugating recombinant proteins to Pseudomonas aeruginosa exotoxin A: a strategy for significantly enhancing immunogenicity of malaria vaccine candidates. Vaccine. 25(20):3923-33.
14. Sachs J, Malaney P (2002) The economic and social burden of malaria. Nature. 415(6872):680-5.
15. Shuler K, Dunham R, and Kanda P. (1992) A simplified method for determination of peptide-protein molar ratios using amino acid analysis. Journal of Immunological Methods. 156:137-149.
16. World Health Organization (WHO). World malaria report 2010. http://www.who.int/malaria/world_malaria_report_2010/en/index.html
17. Wu Y, Przysiecki C, Flanagan E, et al. (2006) Sustained High-titer Antibody Responses

Induced by Conjugating a Malaria Vaccine Candidate to Outer Membrane Protein Complex. Proc. Natl. Acad. Sci. USA. 103: 18243-18248.

18. Wu Y, Ellis RD, Shaffer D, Fontes E, et al. (2008) Phase 1 trial of malaria transmission blocking vaccine candidates Pfs25 and Pvs25 formulated with Montanide ISA 51. PLoS One. 2008 Jul 9;3(7):e2636.

Appendix A: Malaria Comprehension Exam

Vol. Initials: __ __ __
Vol. #: ____________
Screen #: ____________
Date: ___/__/_____

JOHNS HOPKINS
CENTER FOR IMMUNIZATION RESEARCH

Comprehension Assessment

Comprehension Assessment Procedures:

Volunteers will be given the Comprehension Assessment to complete. This Comprehension Assessment consists of **13** open ended or multiple-choice questions. After reading the consent, volunteers will be asked to record their response in the left column on the top page of a two page sealed NCR document. Volunteers are able to refer to the consent while completing the assessment.

See below:

Please answer the following questions with a short answer or by circling the correct answer	
1. What disease is this vaccine being developed to protect against?	**DO NOT WRITE HERE**
2. What are three risks of participating in this study?<br>a)<br>b)<br>c)	
3. What are the direct benefits of participating in this study?	

After the volunteer has completed the assessment:

- Study staff will remove the top page of the sealed NCR document to reveal the bottom page
- Study staff will review the correct answers on the bottom page with the volunteer
- Volunteers are not required to correct their answers or write in correct answers on the left column once the study staff have reviewed the correct answers on the right column
- Questions with more than one correct answer possible will have all correct answers reviewed by the study staff
- Volunteer and study staff will sign document once answers have been reviewed to document understanding of correct answers. **See below:**

1. What disease is this vaccine being developed to protect against?	Malaria
2. What are three risks of participating in this study?	• Injection site reactions<br>• Systemic reactions<br>• Lab abnormalities<br>• Allergic reactions
3. What are the direct benefits of participating in this study?	None

All correct responses have been discussed with me:

Volunteer signature:________________________ Date: ___/___/____

1. What disease is this vaccine being developed to protect against?	**DO NOT WRITE HERE**
2. What are three risks of participating in the study?<br>1.<br><br>2.<br><br>3.	
3. What are the direct benefits of participating in the study?	
4. Is this a vaccine that is currently approved by the FDA for use in the general public?<br>Circle one: YES NO	
5. Why do we not want women who are pregnant to enroll in the study?	
6. Describe three things we will ask you to do while you are in this study.<br>1.	

2.<br><br>3.	
7. What are two reasons you may be removed from the study?<br>1.<br><br>2.	
8. Who should you contact if you have questions about participating in the study?	
9. When can you withdraw from the study?	
10. How long does the study last?	
11. Is Malaria a problem in Baltimore?<br>Circle one: YES NO	
12. Are there unknown risks associated with joining this study?<br>Circle one: YES NO	
13. Of the ____ volunteers enrolled in the study, ____ will get 2 doses of vaccine and ____ will get 3 doses of vaccine. Fill in the correct numbers of volunteers	**DO NOT WRITE HERE**

All correct responses have been discussed with me:

Volunteer signature:________________________ Date: ___/___/____

1. What disease is this vaccine being developed to protect against?	Malaria
2. What are three risks of participating in the study?<br>1.<br><br>2.<br><br>3.	• Injection site reactions<br>• Blood drawing reactions<br>• Systemic symptoms: fever, headache, nausea, fatigue, muscle pains, joint pains<br>• Laboratory abnormalities: low white blood cell count, platelets, abnormal liver or kidney tests
3. What are the direct benefits of participating in the study?	None
4. Is this a vaccine that is currently approved by the FDA for use in the general public?<br>Circle one: YES NO	NO
5. Why do we not want women who are pregnant to enroll in the study?	We don't know the effects of the vaccine on an unborn child
6. Describe three things we will ask you to do while you are in this study.<br>1.<br><br>2.<br><br>3.	• Monitor your temperature and symptoms after vaccination<br>• Get blood draws<br>• Come in for study visits
7. What are two reasons you may be removed from the study?<br>1.<br><br>2.	• For your safety<br>• If you do not make follow-up appointments<br>• At the PI's discretion
8. Who should you contact if you have questions about participating in the study?	• Study PI<br>• Study coordinator<br>• IRB

9. When can you withdraw from the study?	Anytime
10. How long does the study last?	12 months after the last vaccination (14 months for groups 1 and 2; 16 months for group 3)
11. Is Malaria a problem in Baltimore? Circle one: YES NO	NO
12. Are there unknown risks associated with joining this study? Circle one: YES NO	YES
13. Of the ____ volunteers enrolled in the study, ____ will get 2 doses of vaccine and ____ will get 3 doses of vaccine. Fill in the correct numbers of volunteers	30 volunteers will be enrolled. 20 will get 2 doses and 10 will get 3 doses

All correct responses have been discussed with me:

Volunteer signature:________________________ Date: ___/___/____

Appendix B: Schedule of Procedures/Evaluations

Groups 1(a and b)

		Month		0				1	2[3]				3	4	6	9	14
**Procedures**	**Blood Volume[1]**	**Day**			**3**	**7**	**14**	**28**	**56**	**59**	**63**	**70**	**84**	**120**	**180**	**270[5]**	**420**
Complete History/ Physical			X														
Obtain Informed Consent			X														
Interim Clinical Evaluation				X	X	X	X	X	X	X	X	X	X	X	X	X	X
CBC[2]	5 mL		X	X	X	X	X		X	X	X	X					
ALT/ Creatinine	5 mL		X	X	X		X		X	X		X					
Urine dipstick test			X	X		X	X	X	X		X	X	X	X	X		
Urine/serum pregnancy test (females)			X	X			X		X			X					
HIV	5 mL		X														
HbsAg & HCV ELISA	5 mL		X														
**VACCINATION**				**X**					**X**								
Pfs25-EPA ELISA	5 mL			X			X		X			X			X	X	X
Membrane feeding assay[4]	20 mL			X								X					
T & B cell analysis[4]	40 mL			X					X		X	X					X
Daily blood draw			20	75	10	5	15		55	10	45	75			5	5	45
**Cumulative Blood Volume in mL**			20	95	105	110	125		180	190	235	310			315	320	365

[1]Total blood volume to drawn over course of study is 365 mL for Groups 1(a and b) who receive 2 doses, 500 mL for those who get the optional booster dose, 700 mL for Group 2.

With large volume blood draws totals are up to 900 and 1500 mL respectively.
[2]CBC parameters to be assessed for safety: WBC, absolute neutrophil count, hemoglobin, and platelet count.
[3]Days of second and third vaccinations can range according to the specified window, in which case subsequent follow-up should be adjusted accordingly (i.e., Day 1 after second vaccine instead of Day 57, etc.)
[4]Plasma remaining from T and B cell studies will be stored and may be used for transmission blocking assays.
[5] Subjects with exceptionally high antibody titers after the second dose will be offered an optional booster dose. If they accept, their study schedule changes to the one below.

GROUP 1a and 1b High Responders with optional Booster dose

		**Month**	9	10				11	12	15	18	22
**Procedures**	**Blood Volume**[1]	**Day**	270	300	303	307	314	328	356	450	540	660
Complete History/Physical												
Obtain Informed Consent			X									
Interim Clinical Evaluation			X	X	X	X	X	X	X	X	X	X
CBC[2]	5 mL		X	X	X		X					
ALT/Creatinine	5 mL			X	X		X					
Urine dipstick test			X	X		X	X	X	X	X	X	
Urine/serum pregnancy test (females)			X	X			X					
HIV	5 mL											
HbsAg & HCV ELISA	5 mL											
**VACCINATION**				**X**								
Pfs25-EPA ELISA	5 mL		X	X			X	X	X	X	X	X
Membrane feeding assay[4]	20 mL						X		X			
T & B cell analysis[4]	40 mL			X			X					X
Daily blood draw			10	55	10		75	5	25	5	5	45
**Cumulative Blood Volume in mL**			325	380	390	390	465	470	495	500	505	550

Group 2 -Subjects who did not receive Booster dose

		Month		0				1	2[3]				3	4[3]				5	6	9	16
**Procedures**	**Blood Volume[1]**	**Day**							**56**	**59**	**63**	**70**	**84**	**120**	**123**	**127**	**134**	**148**	**175**	**270**	**480**
Complete History/ Physical			X																		
Obtain Informed Consent			X																		
Interim Clinical Evaluation				X	X	X	X	X	X	X	X	X	X	X	X	X	X	X	X	X	X
CBC[2]	5 mL		X	X	X	X	X		X	X	X	X		X	X	X	X				
ALT/ Creatinine	5 mL		X	X	X		X		X	X		X		X	X		X				
Urine dipstick test			X	X		X	X	X	X		X	X	X	X		X	X	X	X	X	
Urine/serum pregnancy test (females)			X	X			X		X			X		X			X				
HIV	5 mL		X																		
HbsAg & HCV ELISA	5 mL		X																		
**VACCINATION**				**X**					**X**					**X**							
Pfs25-EPA ELISA	5 mL			X			X		X			X		X			X	X	X	X	X
Membrane feeding assay[4]	20 mL			X								X					X				
T & B cell analysis[4]	40 mL			X					X		X	X		X			X				X
Daily blood draw			20	75	10	5	15		55	10	45	75		55	10	5	75	5	5	5	45
**Cumulative Blood Volume in mL**			20	95	10<br>5	110	125		18<br>0	190	235	31<br>0		365	375	380	455	460	465	470	515

Group 2 -Subjects who receive booster dose

		**Month**		0				1	2				3	4				5	6	9
**Procedures**	**Blood Volume**[1]	**Day**	Pre	0	3	7	14	28	56	59	63	70	84	120	123	127	134	148	175	270
Complete History/Physical			X																	
Obtain Informed Consent			X																	X
Interim Clinical Evaluation				X	X	X	X	X	X	X	X	X	X	X	X	X	X	X	X	X
CBC[2]	5 mL		X	X	X	X	X		X	X	X	X		X	X	X	X			X
ALT/Creatinine	5 mL		X	X	X		X		X	X		X		X	X		X			
Urine dipstick test			X	X		X	X	X	X		X	X	X	X		X	X	X	X	X
Urine/serum pregnancy test (females)			X	X			X		X			X		X			X			X
HIV	5 mL		X																	
HbsAg & HCV ELISA	5 mL		X																	
**VACCINATION**				**X**					**X**					**X**						
Pfs25-EPA ELISA	5 mL			X			X		X			X		X			X		X	X
Membrane feeding assay[4]	20 mL			X								X					X			
T & B cell analysis[4]	40 mL			X					X		X	X		X		X	X			
Daily blood draw			20	75	10	5	15		55	10	45	75		55	10	5	75		5	10
**Cumulative Blood Volume in mL**			20	95	105	110	125		180	190	235	310		365	375	380	455		460	470

		**Month**	10				11	12	15	18	22
**Procedures**	**Blood Volume**[1]	**Day**	300	303	307	314	328	356	450	540	660
Complete History/Physical											
Obtain Informed Consent											
Interim Clinical Evaluation			X	X	X	X	X	X	X	X	X
CBC[2]	5 mL		X	X		X					
ALT/Creatinine	5 mL		X	X		X					
Urine dipstick test			X		X	X	X	X	X	X	
Urine/serum pregnancy test (females)			X			X					
HIV	5 mL										
HbsAg & HCV ELISA	5 mL										
**VACCINATION**			**X**								
Pfs25-EPA ELISA	5 mL		X			X	X	X	X	X	X
Membrane feeding assay[4]	20 mL					X		X			
T & B cell analysis[4]	40 mL		X			X					X
Daily blood draw			55	10	0	75	5	25	5	5	45
**Cumulative Blood Volume in mL**			525	535	535	610	615	640	645	650	695

Appendix C: Toxicity Table

These tables are modified versions of the FDA Toxicity Grading Scale for healthy adult and adolescent subjects enrolled in Preventive Vaccine Clinical Trials to be used to grade adverse events.

Tables for Clinical Abnormalities

Local Reaction to Injectable Product	Mild (Grade 1)	Moderate (Grade 2)	Severe (Grade 3)	Potentially Life Threatening (Grade 4)
Pain	Does not interfere with activity	Repeated use of non-narcotic pain reliever > 24 hours or interferes with activity	Any use of narcotic pain reliever or prevents daily activity	Emergency room (ER) visit or hospitalization
Erythema/Redness *	2.5 – 5 cm	5.1 – 10 cm	> 10 cm	Necrosis or exfoliative dermatitis
Induration/Swelling **	2.5 – 5 cm and does not interfere with activity	5.1 – 10 cm or interferes with activity	> 10 cm or prevents daily activity	Necrosis

* In addition to grading the measured local reaction at the greatest single diameter, the measurement should be recorded as a continuous variable.
** Induration/Swelling should be evaluated and graded using the functional scale as well as the actual measurement.

Vital Signs * /Systemic adverse events	Mild (Grade 1)	Moderate(Grade 2)	Severe (Grade 3)	Potentially Life Threatening (Grade 4)
Fever (°C) ** (°F) **	38.0 – 38.4 100.4 – 101.1	38.5 – 38.9 101.2 – 102.0	39.0 – 40 102.1 – 104	> 40 > 104
Tachycardia - beats per minute	101 – 115	116 – 130	> 130	ER visit or hospitalization for arrhythmia
Bradycardia - beats per minute***	50 – 54	45 – 49	< 45	ER visit or hospitalization for arrhythmia
Hypertension (systolic) - mm Hg	141 – 150	151 – 155	> 155	ER visit or hospitalization for malignant hypertension
Hypertension (diastolic) - mm Hg	91 – 95	96 – 100	> 100	ER visit or hospitalization for malignant hypertension
Hypotension (systolic) – mm Hg	85 – 89	80 – 84	< 80	ER visit or hospitalization for hypotensive shock
Dyspnea	Dyspnea on exertion	Dyspnea with normal activity	Dyspnea at rest	Requires hospitalization

Vital Signs * /Systemic adverse events	Mild (Grade 1)	Moderate(Grade 2)	Severe (Grade 3)	Potentially Life Threatening (Grade 4)
Nausea/vomiting	No interference with activity or 1 – 2 episodes/24 hours	Some interference with activity or > 2 episodes/24 hours	Prevents daily activity, requires outpatient IV hydration	ER visit or hospitalization for hypotensive shock
Diarrhea	2 – 3 loose stools or < 400 gms/24 hours	4 – 5 stools or 400 – 800 gms/24 hours	6 or more watery stools or > 800gms/24 hours or requires outpatient IV hydration	ER visit or hospitalization
Headache	No interference with activity	Repeated use of non-narcotic pain reliever > 24 hours or some interference with activity	Significant; any use of narcotic pain reliever or prevents daily activity	ER visit or hospitalization
Fatigue/Malaise	No interference with activity	Repeated use of non-narcotic pain reliever > 24 hours or some interference with activity	any use of narcotic pain reliever or prevents daily activity	ER visit or hospitalization
Myalgia	No interference with activity	Repeated use of non-narcotic pain reliever > 24 hours or some interference with activity	any use of narcotic pain reliever or prevents daily activity	ER visit or hospitalization
Arthralgia	No interference with activity	Repeated use of non-narcotic pain reliever > 24 hours or some interference with activity	any use of narcotic pain reliever or prevents daily activity	ER visit or hospitalization
Urticaria	No interference with activity	Requiring PO or topical treatment > 24 hours or IV medications or steroids for ≤24 hours	Requiring IV medication or steroids for >24 hours	ER visit or hospitalization

* Subject should be at rest for all vital sign measurements.
** Oral temperature; no recent hot or cold beverages or smoking.
*** When resting heart rate is between 60 – 100 beats per minute. Use clinical judgment when characterizing bradycardia among some healthy subject populations, for example, conditioned athletes.

Serum*	Mild (Grade 1)	Moderate (Grade 2)	Severe (Grade 3)	Potentially Life Threatening (Grade 4)**
Sodium – Hyponatremia mEq/L	132 – 134	130 – 131	125 – 129	< 125
Sodium – Hypernatremia mEq/L	144 – 145	146 – 147	148 – 150	> 150
Potassium – Hyperkalemia mEq/L	5.1 – 5.2	5.3 – 5.4	5.5 – 5.6	> 5.6
Potassium – Hypokalemia mEq/L	3.5 – 3.6	3.3 – 3.4	3.1 – 3.2	< 3.1
Glucose – Hypoglycemia mg/dL	65 – 69	55 – 64	45 – 54	< 45
Glucose – Hyperglycemia<br>Fasting – mg/dL<br>Random – mg/dL	100 – 110<br>110 – 125	111 – 125<br>126 – 200	>125<br>>200	Insulin requirements or hyperosmolar coma
Blood Urea Nitrogen BUN mg/dL	23 – 26	27 – 31	> 31	Requires dialysis
Creatinine – mg/dL	1.5 – 1.7	1.8 – 2.0	2.1 – 2.5	> 2.5 or requires dialysis
Calcium – hypocalcemia mg/dL	8.0 – 8.4	7.5 – 7.9	7.0 – 7.4	< 7.0
Calcium – hypercalcemia mg/dL	10.5 – 11.0	11.1 – 11.5	11.6 – 12.0	> 12.0
Magnesium – hypomagnesemia mg/dL	1.3 – 1.5	1.1 – 1.2	0.9 – 1.0	< 0.9
Phosphorous – hypophosphatemia mg/dL	2.3 – 2.5	2.0 – 2.2	1.6 – 1.9	< 1.6
CPK – mg/dL	1.25 – 1.5 x ULN***	1.6 – 3.0 x ULN	3.1 –10 x ULN	> 10 x ULN
Albumin – Hypoalbuminemia g/dL	2.8 – 3.1	2.5 – 2.7	< 2.5	--
Total Protein – Hypoproteinemia g/dL	5.5 – 6.0	5.0 – 5.4	< 5.0	--
Alkaline phosphate increase by factor	1.1 – 2.0 x ULN	2.1 – 3.0 x ULN	. 3.1 – 10 x ULN	> 10 x ULN
Liver Function Tests – ALT, AST<br>increase by factor	1.1 – 2.5 x ULN	2.6 – 5.0 x ULN	5.1 – 10 x ULN	> 10 x ULN
Bilirubin – when accompanied<br>by any increase in Liver Function Test<br>increase by factor	1.1 – 1.25 x ULN	1.26 – 1.5 x ULN	1.51 – 1.75 x ULN	> 1.75 x ULN

* The laboratory values provided in the tables serve as guidelines and are dependent upon institutional normal parameters. Institutional normal reference ranges should be provided to demonstrate that they are appropriate.
** The clinical signs or symptoms associated with laboratory abnormalities might result in characterization of the laboratory abnormalities as Potentially Life Threatening (Grade 4). For example, a low sodium value that falls within a grade 3 parameter (125-129 mE/L) should be recorded as a grade 4 hyponatremia event if the subject had a new seizure associated with the low sodium value.
***ULN" is the upper limit of the normal range.

Hematology *	Mild (Grade 1)	Moderate (Grade 2)	Severe (Grade 3)	Potentially Life Threatening (Grade 4)
Hemoglobin (Female) - gm/dL	10.0 -11.2	8.5 - 9.9	7.5 – 8.4	< 7.5
Hemoglobin (Male) - gm/dL	12.0 – 13.0	10.0 - 11.9	8.0 - 9.9	< 8.0
WBC Increase - cell/mm^3	10,800 – 15,000	15,001 – 20,000	20,001 – 25, 000	> 25,000
WBC Decrease - cell/mm^3	2,500-3500	1,500 – 2499	1,000 – 1,499	< 1,000
Lymphocytes Decrease - cell/mm^3	750 – 1,000	500 – 749	250 – 499	< 250
Neutrophils Decrease - cell/mm^3	1000-1499	750-999	500 – 749	< 500
Eosinophils - cell/mm^3	650 – 1500	1501 - 5000	> 5000	Hypereosinophilic
Platelets Decreased - cell/mm^3	125000-140000	100000-124000	25,000 – 99000	< 25,000
PT – increase by factor (prothrombin time)	1.0 – 1.10 x ULN**	. 1.11 – 1.20 x ULN	1.21 – 1.25 x ULN	> 1.25 ULN
PTT – increase by factor (partial thromboplastin time)	1.0 – 1.10 x ULN	1.11 – 1.20 x ULN	1.21 – 1.25 x ULN	> 1.5 x ULN
Fibrinogen increase - mg/dL	400 – 500	501 – 600	> 600	--

* The laboratory values provided in the tables serve as guidelines and are dependent upon institutional normal parameters. Institutional normal reference ranges should be provided to demonstrate that they are appropriate.
** "ULN" is the upper limit of the normal range.

Urine*	Mild (Grade 1)	Moderate (Grade 2)	Severe (Grade 3)	Potentially Life Threatening (Grade 4)
Protein	Trace	1+	2+	Hospitalization or dialysis
Glucose	Trace	1+	2+	Hospitalization for hyperglycemia
Blood (microscopic) – red blood cells per high power field (rbc/hpf)	1 - 10	11 – 50	> 50 and/or gross blood	Hospitalization or packed red blood cells (PRBC) transfusion

* The laboratory values provided in the tables serve as guidelines and are dependent upon institutional normal parameters. Institutional normal reference ranges should be provided to demonstrate that they are appropriate.
