# Supplementary material for: Safety and Immunogenicity of Pfs25-EPA/Alhydrogel®, a Transmission Blocking Vaccine against Plasmodium falciparum: An Open Label Study in Malaria Naïve Adults
Source: PLoS One. 2016 Oct 17;11(10):e0163144. doi: 10.1371/journal.pone.0163144 (PMC5066979; doi:10.1371/journal.pone.0163144)
Supplement: S1 Table — (DOCX) [file pone.0163144.s004.docx]

**S2 Table. ELISA titer, IFA titer, and TRA of sera from selected volunteers**

| Subject ID | **D314 serum** | | | | **D356 serum** | | | |
| --- | --- | --- | --- | --- | --- | --- | --- | --- |
|  | ELISA Titer^a^ | IFA^b^ 1:500 | IFA^b^ 1:1000 | TRA^c^ | ELISA Titer^a^ | IFA^b^ 1:500 | IFA^b^ 1:1000 | TRA^c^ |
| 20 | 434 | Positive | Positive | 95.8/94.9 | 149 | Positive | Positive | 81.5/84.2 |
| 25 | 247 | Positive | Positive | 77.3/83.1 | 77 | Positive | Negative | 40.3/45.9 |
| 29 | 86 | Not tested | Positive | 63.2/49.9 | 41 | Not tested | Negative | 34.9/54.1 |

**^a^** Value indicates anti-Pfs25 specific IgG levels in µg/mL

^b^ Value indicate dilutions of the antiserum used in the assay

^c^ Value indicates TRA in 2 independent SMFAs
