# Supplementary material for: Safety and Immunogenicity of Pfs25-EPA/Alhydrogel®, a Transmission Blocking Vaccine against Plasmodium falciparum: An Open Label Study in Malaria Naïve Adults
Source: PLoS One. 2016 Oct 17;11(10):e0163144. doi: 10.1371/journal.pone.0163144 (PMC5066979; doi:10.1371/journal.pone.0163144)
Supplement: S2 Table — (DOCX) [file pone.0163144.s005.docx]

**S2 Table. SMFA Results with Individual Test Serum at Each Time Point**

| **D70 Sera with Individual D0 Sera as Control** | | | | | | | | | | | | | |
| --- | --- | --- | --- | --- | --- | --- | --- | --- | --- | --- | --- | --- | --- |
|  |  | **Experiment 1** | | | | | | **Experiment 2** | | | | | |
|  |  | **Control** | | **Test** | | | | **Control** | | **Test** | | | |
| **Group** | **Subject** | **Infected/Dissected** | **Mean Ooc** | **Infected/Dissected** | **Mean Ooc** | **TBA** | **TRA** | **Infected/Dissected** | **Mean Ooc** | **Infected/Dissected** | **Mean Ooc** | **TBA** | **TRA** |
| **G1a,**  **8 µg Pfs25** | 01 | 24/24 | 56.46 | 25/25 | 44.32 | 0.0 | 21.5 | 26/26 | 19.85 | 29/29 | 17.10 | 0.0 | 13.8 |
|  | 02 | 19/19 | 47.47 | 21/21 | 58.57 | 0.0 | -23.4 | 22/22 | 40.55 | 21/22 | 33.32 | 4.6 | 17.8 |
|  | 03 | 30/30 | 30.37 | 21/31 | 1.90 | 32.3 | 93.7 | 19/19 | 55.53 | 18/18 | 8.56 | 0.0 | 84.6 |
|  | 04 | 25/26 | 27.85 | 24/24 | 37.79 | -4.0 | -35.7 | 27/27 | 11.26 | 25/25 | 11.44 | 0.0 | -1.6 |
|  | 05 | 26/26 | 27.92 | 26/26 | 27.15 | 0.0 | 2.8 | 23/24 | 20.38 | 30/31 | 22.42 | -1.0 | -10.0 |
| **G1b, 16 µg Pfs25** | 06 | 29/31 | 36.30 | 25/25 | 31.60 | -3.5 | 13.0 | 18/18 | 66.39 | 19/19 | 51.79 | 0.0 | 22.0 |
|  | 07 | 29/29 | 42.00 | 30/30 | 42.77 | 0.0 | -1.8 | 19/19 | 71.32 | 21/21 | 41.33 | 0.0 | 42.0 |
|  | 08 | 26/26 | 60.77 | 26/26 | 63.46 | 0.0 | -4.4 | 22/22 | 39.45 | 22/22 | 37.00 | 0.0 | 6.2 |
|  | 09 | 24/24 | 39.00 | 27/27 | 27.74 | 0.0 | 28.9 | 31/31 | 21.45 | 27/29 | 12.07 | 6.9 | 43.7 |
| **G2,**  **47 µg Pfs25** | 11 | 22/26 | 26.81 | 29/29 | 29.07 | -18.2 | -8.4 | 27/27 | 15.15 | 28/28 | 16.68 | 0.0 | -10.1 |
|  | 12 | 30/31 | 40.68 | 26/26 | 50.46 | -3.3 | -24.1 | 28/29 | 21.07 | 24/25 | 21.84 | 0.6 | -3.7 |
|  | 13 | 27/28 | 42.46 | 28/29 | 38.55 | -0.1 | 9.2 | 18/18 | 51.06 | 19/19 | 48.79 | 0.0 | 4.4 |
|  | 15 | 24/24 | 44.46 | 28/28 | 43.21 | 0.0 | 2.8 | 27/27 | 17.15 | 28/28 | 20.39 | 0.0 | -18.9 |
|  | 16 | 24/25 | 47.60 | 27/28 | 25.21 | -0.5 | 47.0 | 28/28 | 24.43 | 25/25 | 16.36 | 0.0 | 33.0 |
|  | 17 | 25/25 | 47.40 | 28/28 | 56.75 | 0.0 | -19.7 | 22/22 | 54.23 | 23/23 | 34.00 | 0.0 | 37.3 |
|  | 18 | 20/20 | 56.85 | 26/27 | 16.89 | 3.7 | 70.3 | 21/21 | 39.24 | 27/27 | 11.78 | 0.0 | 70.0 |
|  | 19 | 25/25 | 52.16 | 26/26 | 46.81 | 0.0 | 10.3 | 22/25 | 34.40 | 23/23 | 25.04 | -13.6 | 27.2 |
|  | 20 | 24/25 | 24.20 | 29/31 | 15.03 | 2.6 | 37.9 | 20/20 | 13.95 | 20/21 | 13.76 | 4.8 | 1.4 |
|  | 22 | 27/27 | 50.67 | 20/20 | 25.20 | 0.0 | 50.3 | 35/35 | 38.20 | 40/42 | 28.40 | 4.8 | 25.6 |
|  | 23 | 27/29 | 67.76 | 24/25 | 32.84 | -3.1 | 51.5 | 41/42 | 38.62 | 37/38 | 23.55 | 0.3 | 39.0 |
|  | 24 | 28/29 | 40.41 | 26/26 | 29.88 | -3.6 | 26.1 | 25/26 | 21.81 | 29/29 | 19.83 | -4.0 | 9.1 |
|  | 25 | 30/30 | 52.20 | 25/25 | 41.48 | 0.0 | 20.5 | 25/25 | 20.36 | 25/25 | 26.04 | 0.0 | -27.9 |
|  | 27 | 24/25 | 42.32 | 23/26 | 16.23 | 7.9 | 61.7 | 33/34 | 19.06 | 33/39 | 6.72 | 12.8 | 64.8 |
|  | 28 | 25/25 | 31.48 | 27/29 | 26.38 | 6.9 | 16.2 | 34/36 | 17.50 | 24/31 | 4.13 | 18.0 | 76.4 |
|  | 29 | 26/27 | 41.74 | 22/23 | 24.13 | 0.7 | 42.2 | 30/31 | 17.81 | 35/37 | 13.62 | 2.3 | 23.5 |
|  | 30 | 30/32 | 30.47 | 25/25 | 34.96 | -6.7 | -14.7 | 33/33 | 16.82 | 27/32 | 11.72 | 15.6 | 30.3 |

| **D134 Sera with Individual D0 Sera as Control** | | | | | | | | | | | | | |
| --- | --- | --- | --- | --- | --- | --- | --- | --- | --- | --- | --- | --- | --- |
|  |  | **Experiment 1** | | | | | | **Experiment 2** | | | | | |
|  |  | **Control** | | **Test** | | | | **Control** | | **Test** | | | |
| **Group** | **Subject** | **Infected/Dissected** | **Mean Ooc** | **Infected/Dissected** | **Mean Ooc** | **TBA** | **TRA** | **Infected/Dissected** | **Mean Ooc** | **Infected/Dissected** | **Mean Ooc** | **TBA** | **TRA** |
| **G2,**  **47 µg Pfs25** | 11 | 23/23 | 68.61 | 25/25 | 63.84 | 0.0 | 7.0 | 25/26 | 16.42 | 24/26 | 16.04 | 3.9 | 2.3 |
|  | 12 | 25/25 | 58.44 | 23/23 | 83.30 | 0.0 | -42.6 | 23/24 | 20.04 | 25/25 | 19.28 | -4.2 | 3.8 |
|  | 13 | 24/24 | 11.58 | 25/26 | 8.85 | 3.9 | 23.6 | 27/30 | 25.93 | 25/25 | 39.60 | -10.0 | -52.7 |
|  | 15 | 24/24 | 82.58 | 24/24 | 62.33 | 0.0 | 24.5 | 26/26 | 18.46 | 25/26 | 10.19 | 3.9 | 44.8 |
|  | 16 | 22/24 | 61.50 | 25/25 | 45.64 | -8.3 | 25.8 | 21/24 | 5.21 | 23/25 | 10.60 | -4.3 | -103.5 |
|  | 17 | 27/28 | 14.50 | 26/28 | 5.57 | 7.1 | 61.6 | 27/28 | 26.29 | 28/28 | 22.36 | -4.3 | 15.0 |
|  | 20 | 24/24 | 51.29 | 23/24 | 21.08 | 4.2 | 58.9 | 23/25 | 7.84 | 13/26 | 1.27 | 42.0 | 83.8 |
|  | 22 | 24/24 | 71.88 | 25/25 | 55.92 | 0.0 | 22.2 | 24/24 | 15.92 | 24/26 | 14.65 | 7.7 | 7.9 |
|  | 23 | 24/24 | 72.04 | 24/25 | 37.48 | 4.0 | 48.0 | 23/24 | 10.38 | 24/24 | 16.54 | -4.2 | -59.4 |
|  | 24 | 26/26 | 12.04 | 27/28 | 6.11 | 3.6 | 49.3 | 26/26 | 37.42 | 26/27 | 28.52 | 3.6 | 23.8 |
|  | 25 | 26/26 | 24.42 | 27/27 | 13.85 | 0.0 | 43.3 | 28/28 | 49.50 | 25/25 | 48.68 | 0.0 | 1.7 |
|  | 27 | 25/27 | 12.37 | 25/25 | 6.04 | -7.4 | 51.2 | 26/27 | 24.59 | 25/27 | 33.11 | 3.7 | -34.6 |
|  | 28 | 22/23 | 18.43 | 24/25 | 5.68 | 4.0 | 66.2 | 22/27 | 8.04 | 25/26 | 28.15 | -14.7 | -250.3 |
|  | 29 | 24/25 | 51.52 | 24/26 | 16.85 | 3.5 | 67.3 | 23/23 | 11.52 | 24/24 | 5.38 | 0.0 | 53.4 |
|  | 30 | 24/25 | 12.24 | 25/26 | 11.15 | -0.2 | 8.9 | 24/26 | 37.00 | 25/26 | 42.08 | -4.0 | -13.7 |

| **D314 Sera with a Pooled D0 Serum as Control** | | | | | | | | | | | | | |
| --- | --- | --- | --- | --- | --- | --- | --- | --- | --- | --- | --- | --- | --- |
|  |  | **Experiment 1** | | | | | | **Experiment 2** | | | | | |
|  |  | **Control** | | **Test** | | | | **Control** | | **Test** | | | |
| **Group** | **Subject** | **Infected/Dissected** | **Mean Ooc** | **Infected/Dissected** | **Mean Ooc** | **TBA** | **TRA** | **Infected/Dissected** | **Mean Ooc** | **Infected/Dissected** | **Mean Ooc** | **TBA** | **TRA** |
| **G1a,**  **8 µg Pfs25** | 3 | 20/20 | 112.65 | 18/23 | 3.87 | 21.7 | 96.6 | 21/21 | 63.33 | 18/23 | 7.57 | 21.7 | 88.1 |
| **G2,**  **47 µg Pfs25** | 11 | 20/20 | 112.65 | 20/21 | 39.67 | 4.8 | 64.8 | 21/21 | 63.33 | 21/21 | 25.33 | 0.0 | 60.0 |
|  | 12 |  |  | 21/21 | 46.86 | 0.0 | 58.4 |  |  | 21/22 | 40.86 | 4.6 | 35.5 |
|  | 13 |  |  | 19/21 | 4.71 | 9.5 | 95.8 |  |  | 22/23 | 45.30 | 4.4 | 28.5 |
|  | 15 |  |  | 20/21 | 39.10 | 4.8 | 65.3 |  |  | 21/22 | 43.18 | 4.6 | 31.8 |
|  | 20 |  |  | 18/22 | 4.77 | 18.2 | 95.8 |  |  | 19/28 | 3.21 | 32.1 | 94.9 |
|  | 22 |  |  | 5/22 | 0.45 | 77.3 | 99.6 |  |  | 21/22 | 28.32 | 4.6 | 55.3 |
|  | 24 |  |  | 22/22 | 21.82 | 0.0 | 80.6 |  |  | 22/25 | 15.84 | 12.0 | 75.0 |
|  | 25 |  |  | 19/21 | 25.62 | 9.5 | 77.3 |  |  | 22/25 | 10.72 | 12.0 | 83.1 |
|  | 28 |  |  | 22/22 | 24.91 | 0.0 | 77.9 |  |  | 22/23 | 9.61 | 4.4 | 84.8 |
|  | 29 |  |  | 22/22 | 41.50 | 0.0 | 63.2 |  |  | 24/24 | 31.75 | 0.0 | 49.9 |
|  | 30 |  |  | 18/20 | 39.90 | 10.0 | 64.6 |  |  | 25/27 | 32.89 | 7.4 | 48.1 |

| **D356 Sera with a Pooled D0 Serum as Control** | | | | | | | | | | | | | |
| --- | --- | --- | --- | --- | --- | --- | --- | --- | --- | --- | --- | --- | --- |
|  |  | **Experiment 1** | | | | | | **Experiment 2** | | | | | |
|  |  | **Control** | | **Test** | | | | **Control** | | **Test** | | | |
| **Group** | **Subject** | **Infected/Dissected** | **Mean Ooc** | **Infected/Dissected** | **Mean Ooc** | **TBA** | **TRA** | **Infected/Dissected** | **Mean Ooc** | **Infected/Dissected** | **Mean Ooc** | **TBA** | **TRA** |
| **G1a,**  **8 µg Pfs25** | 3 | 21/21 | 64.19 | 20/21 | 11.29 | 4.8 | 82.4 | 20/21 | 25.95 | 14/21 | 2.48 | 29.8 | 90.5 |
| **G2,**  **47 µg Pfs25** | 11 | 21/21 | 64.19 | 21/21 | 49.86 | 0.0 | 22.3 | 20/21 | 25.95 | 20/21 | 18.33 | 0.0 | 29.4 |
|  | 12 |  |  | 20/21 | 47.38 | 4.8 | 26.2 |  |  | 20/20 | 27.70 | -5.3 | -6.7 |
|  | 13 |  |  | 21/21 | 59.95 | 0.0 | 6.6 |  |  | 22/22 | 26.55 | -5.3 | -2.3 |
|  | 15 |  |  | 21/21 | 58.86 | 0.0 | 8.3 |  |  | 20/20 | 36.20 | -5.3 | -39.5 |
|  | 20 |  |  | 21/21 | 11.90 | 0.0 | 81.5 |  |  | 18/21 | 4.10 | 9.8 | 84.2 |
|  | 22 |  |  | 21/21 | 52.67 | 0.0 | 18.0 |  |  | 21/21 | 21.95 | -5.3 | 15.4 |
|  | 24 |  |  | 21/21 | 38.24 | 0.0 | 40.4 |  |  | 21/22 | 16.68 | -0.5 | 35.7 |
|  | 25 |  |  | 21/21 | 38.33 | 0.0 | 40.3 |  |  | 22/22 | 14.05 | -5.3 | 45.9 |
|  | 29 |  |  | 21/21 | 41.76 | 0.0 | 34.9 |  |  | 19/21 | 11.90 | 4.8 | 54.1 |
|  | 30 |  |  | 20/21 | 43.86 | 4.8 | 31.7 |  |  | 19/21 | 24.00 | 4.8 | 7.5 |
